# Functional characteristics of hub and wave-initiator cells in β cell networks

**DOI:** 10.1016/j.bpj.2023.01.039

**Published:** 2023-02-02

**Authors:** Marko Šterk, Jurij Dolenšek, Maša Skelin Klemen, Lidija Križančić Bombek, Eva Paradiž Leitgeb, Jasmina Kerčmar, Matjaž Perc, Marjan Slak Rupnik, Andraž Stožer, Marko Gosak

**Affiliations:** 1 Faculty of Natural Sciences and Mathematics, University of Maribor, Maribor, Slovenia; 2 Faculty of Medicine, University of Maribor, Maribor, Slovenia; 3 Department of Medical Research, China Medical University Hospital, China Medical University, Taichung, Taiwan; 4 Alma Mater Europaea, Maribor, Slovenia; 5 Complexity Science Hub Vienna, Vienna, Austria; 6 Department of Physics, Kyung Hee University, Dongdaemun-gu, Seoul, Republic of Korea; 7 Center for Physiology and Pharmacology, Medical University of Vienna, Vienna, Austria

## Abstract

Islets of Langerhans operate as multicellular networks in which several hundred β cells work in synchrony to produce secretory pulses of insulin, a hormone crucial for controlling metabolic homeostasis. Their collective rhythmic activity is facilitated by gap junctional coupling and affected by their functional heterogeneity, but the details of this robust and coordinated behavior are still not fully understood. Recent advances in multicellular imaging and optogenetic and photopharmacological strategies, as well as in network science, have led to the discovery of specialized β cell subpopulations that were suggested to critically determine the collective dynamics in the islets. In particular hubs, i.e., β cells with many functional connections, are believed to significantly enhance communication capacities of the intercellular network and facilitate an efficient spreading of intercellular Ca^2+^ waves, whereas wave-initiator cells trigger intercellular signals in their cohorts. Here, we determined Ca^2+^ signaling characteristics of these two β cell subpopulations and the relationship between them by means of functional multicellular Ca^2+^ imaging in mouse pancreatic tissue slices in combination with methods of complex network theory. We constructed network layers based on individual Ca^2+^ waves to identify wave initiators, and functional correlation-based networks to detect hubs. We found that both cell types exhibit a higher-than-average active time under both physiological and supraphysiological glucose concentrations, but also that they differ significantly in many other functional characteristics. Specifically, Ca^2+^ oscillations in hubs are more regular, and their role appears to be much more stable over time than for initiator cells. Moreover, in contrast to wave initiators, hubs transmit intercellular signals faster than other cells, which implies a stronger intercellular coupling. Our research indicates that hubs and wave-initiator cell subpopulations are both natural features of healthy pancreatic islets, but their functional roles in principle do not overlap and should thus not be considered equal.

## Significance

Healthy pancreatic islets contain hundreds of β cells that operate in synchrony to secrete pulses of insulin and thereby ensure metabolic homeostasis. The collective activity within these functional syncytia is influenced by two subpopulations of β cells, namely hubs and wave-initiator cells. The latter operate as triggers of intercellular Ca^2+^ signals that synchronize the cells, while the former facilitate their spreading due to their exceptional role in the multicellular network. Here, we determine signaling characteristics of β cell populations while they are still embedded in pancreatic tissue, assess their potential overlap, and their persistency over time. Understanding how the collective rhythmicity is established within heterogeneous cellular subpopulations is of paramount importance also to assess the changes associated with the pathogenesis of diabetes.

## Introduction

Decoding cellular responses to changes in the environment is of fundamental importance to our understanding of living systems (1). While in the past most of the studies focused on isolated cells or population averages, the scope is nowadays shifting toward networked cell populations. This approach is also being applied to the insulin-secreting β cells from pancreatic islets of Langerhans. These microorgans orchestrate oscillations in the circulating insulin with a period of about 3–15 min, which is crucial for maintaining normal homeostasis of glucose and other nutrients (2,3). β Cells are the most prevalent cell type within islets (4,5,6), and are nutrient-sensing units that respond to glucose stimulation with two distinctive phases (7,8,9,10,11,12). Following stimulation and after a time needed for β cells to metabolize glucose, β cells respond with an initial transient increase in intracellular Ca^2+^ concentration ([Ca^2+^]_IC_). This phase is typically referred to as the first phase of response. Afterward, β cells exhibit repetitive [Ca^2+^]_IC_ oscillations that persist during the course of stimulation, and this phase is named the second or the sustained phase of response. Both phases were found to be strongly glucose dependent (11,13,14). Most importantly, β cells do not interact only with their environment but also among themselves. They are strongly electrically coupled and form a functional syncytium. Cell-to-cell interactions encompass direct electrical coupling through gap junctions composed of connexin36, as well as by paracrine, autocrine, and juxtacrine signaling (15,16,17,18,19). Intercellular coupling is essential for the coordination of cellular responses through which insulin is released in proportion to stimulation and metabolic demands (17,20,21,22,23). If, however, cell-to-cell communication is impaired, the coherent patterns of cellular activity are abolished, leading to dysregulated plasma insulin oscillations and to glucose intolerance (21,24), as observed in numerous models of obesity and diabetes mellitus (20,25,26,27,28).

Early studies assumed that β cell populations are rather homogeneous, but the subsequent functional analyses have discovered a remarkable degree of heterogeneity that manifested itself on the transcriptomic (29,30,31,32,33), metabolic (34), electrophysiological (31,32,35,36,37,38), calcium (11,35,39), and secretory (40,41) levels. This heterogeneity has important functional implications, as the presence of specialized subpopulations of β cells probably has a significant impact on how the cells respond to changes in the biochemical composition of their environment, on their collective activity, and consequently on insulin release (7,42).

In recent years, advances in multicellular imaging of [Ca^2+^]_IC_ accompanied by network analyses have become an indispensable tool for investigating how cellular heterogeneity within islets affects their collective activity and function (27,43,44,45,46,47,48,49,50). Historically, network analysis focused mainly on the response during the sustained glucose stimulation. It is now generally accepted that, during this phase a subpopulation of cells (termed hub cells) shows a disproportionally high number of functional connections with other cells (35,43,44,46,49,51,52,53,54). A relatively high fraction of hub cells, short internodal distances, and highly clustered organization, all characteristics of broad-scale small-world networks, are believed to enhance communication capacity and robustness to perturbations within islets (7,44,55). The role of hub cells and their impact on islet function has been extensively debated, offering opposing views on the matter. In principle, electrophysiologists are in general skeptic about the concept of exceptional cells, whereas imaging and molecular biology experts are vigorously defending these ideas (46,53,56,57,58,59,60). Currently, the presence of hub cells in β cell functional networks is well acknowledged, but their exact functional roles have yet to be determined. It is important to note that broad-scale small-world functional networks with hub cells can arise due to heterogeneous nearest-neighbor coupling of heterogeneous β cells that are synchronized by propagating intercellular waves, without the need for physical long-range connections or small-world networks (61). Furthermore, [Ca^2+^]_IC_ wave analyses along with photopharmacological interventions identified subpopulations of cells that rank first during a particular intercellular wave, i.e., wave-initiator cells. In the literature, these cells have often been termed pacemaker cells (43,44). Since some authors suggest that the term pacemaker should be reserved for cells that display an intrinsic oscillatory behavior largely independent of the prevailing conditions and are necessary for oscillations to occur in other cells (which β cells do not), in this work, we refer to these cells as wave initiators (42). In addition, since there are different types of oscillations in the islets that are possibly all synchronized by intercellular waves, we wish to underline that, in this work, we focused on the so-called fast [Ca^2+^]_IC_ activity and their corresponding intercellular waves (6,62). This subpopulation of cells elevate their [Ca^2+^]_IC_ earlier than the rest of the cells during the course of wave spreading and have been reported to be characterized by elevated excitability levels, increased glucokinase activity, and higher-than-average natural frequencies (13,63,64,65). Importantly, the wave-initiator cells should not be confused with another subpopulation, i.e., first responder cells. These cells were identified in the initial transient phase when the cells respond to stimulatory glucose levels and differ in principle from the cells that trigger the repetitive intercellular waves during sustained activity. The first responder cells have been shown to display high excitability (7,44,59) and large heterogeneity that is glucose dependent (11,66). Thus, the existence and importance of β cell subpopulations are now generally acknowledged, and we are starting to unveil the relative contributions of these subpopulations to collective β cell activity in different phases. However, the studies described above focused on particular subpopulations and, to the best of our knowledge, none characterized the subpopulations simultaneously, thus hampering our understanding of their complex interactions. In an attempt to unify seemingly opposing and partial views on β cell heterogeneity, in this study we applied complex network-based analyses in combination with high-resolution multicellular confocal [Ca^2+^]_IC_ imaging in acute mouse pancreas tissue slices. We meticulously describe the Ca^2+^ signaling characteristics of different β cell subpopulations and evaluate their overlap as well as their temporal stability, with a special emphasis on the recently debated relationship or overlap between hub and wave-initiator cells.

## Materials and methods

### Experimental protocol

The study was conducted in strict accordance with all national and European legislation (Directive 63/2010/EU) and recommendations on care and work with laboratory animals and approved by the Administration for Food Safety, Veterinary Sector and Plant Protection of the Republic of Slovenia (approval nos. U34401-12/2015/3 and U34401-35/2018-2). Acute pancreas tissue slices were prepared from nine male NMRI mice aged 2–5 months, as described previously (39,67,68). In brief, after sacrificing the animals by cervical dislocation, the abdominal cavity was accessed via laparotomy. The common bile duct was clamped distally at the major duodenal papilla. The pancreas was injected at the proximal end with 1.9% low-melting-point agarose (Lonza Rockland, ME), which was dissolved in extracellular solution (ECS) (consisting of 125 mM NaCl, 26 mM NaHCO_3_, 6 mM glucose, 6 mM lactic acid, 3 mM myo-inositol, 2.5 mM KCl, 2 mM Na-pyruvate, 2 mM CaCl_2_, 1.25 mM NaH_2_PO_4_, 1 mM MgCl_2_, 0.5 mM ascorbic acid), and maintained at 40°C. Following injection, the pancreas was cooled with the ice-cold ECS, extracted from the animal, placed into a petri dish containing ice-cold ECS, and cut into approximately 100 mm^3^ pieces, which were afterward embedded into agarose and cut with a vibratome (VT 1000 S, Leica Biosystems, Deer Park, IL) into 140 *μ*m thick slices. Throughout the entire procedure, the ECS was continuously bubbled with a gas mixture of 95% O_2_ and 5% CO_2_ at barometric pressure to ensure a pH of 7.4 and proper oxygenation. During cutting slices were gently collected and transferred into a 100 mm petri dish containing 40 mL of HEPES-buffered saline (HBS) (consisting of 150 mM NaCl, 10 mM HEPES, 6 mM glucose, 5 mM KCl, 2 mM CaCl_2_, 1 mM MgCl_2_; titrated to pH 7.4 with 1 M NaOH) with 6 mM glucose at room temperature until being dyed. For dye loading, the slices were transferred into a 5 mL petri dish containing 6 *μ*M Calbryte 520 AM (AAT Bioquest, Sunnyvale, CA), 0.03% Pluronic F-127 (w/v), and 0.12% dimethyl sulfoxide (v/v) dissolved in HBS for 50 min at room temperature on an orbital shaker. Unless stated otherwise, all chemicals were obtained from Sigma-Aldrich (St. Louis, MO).

Individual stained slices were placed into the recording chamber for microscopy, continuously perfused with carbogenated ECS containing 6 mM glucose at 37°C. Perfusate was changed manually to ECS containing 8 or 12 mM glucose concentration at 37°C for 40 or 20 min, respectively, to stimulate β cells. The slice was reintroduced to the perfusate containing 6 mM glucose in ECS for at least 15 min after stimulation. For confocal functional multicellular Ca^2+^ imaging, we used a Leica TCS SP5 AOBS Tandem II upright confocal microscope system with a Leica HCX APO L water immersion objective (20×, NA = 1.0). Fluorophore was excited two to three cell layers deep in the tissue (39) with a 488 nm argon laser to avoid damaged cells at the slice surface. The fluorescence was detected with a Leica HyD hybrid detector in the range of 500–700 nm (all from Leica Microsystems, Wetzlar, Germany). Before and after the time series, high-resolution (1024 × 1024 pixels) images were acquired to assess possible sample motions. The resolution of the image series was 512 × 512 pixels at 10 Hz allowing discrimination of individual [Ca^2+^]_IC_ oscillation at single-cell resolution. Individual cells in islets were manually selected for each time series without motion artifacts as regions of interest (ROIs) (46) and exported using custom software (ImageFiltering, copyright Denis Špelič). Further analysis with in-house MATLAB scripts included a combination of linear and exponential fitting to eliminate photobleaching effects (39).

### Ca^2+^ signal processing and analysis of β cell activity

**Figure 1:**
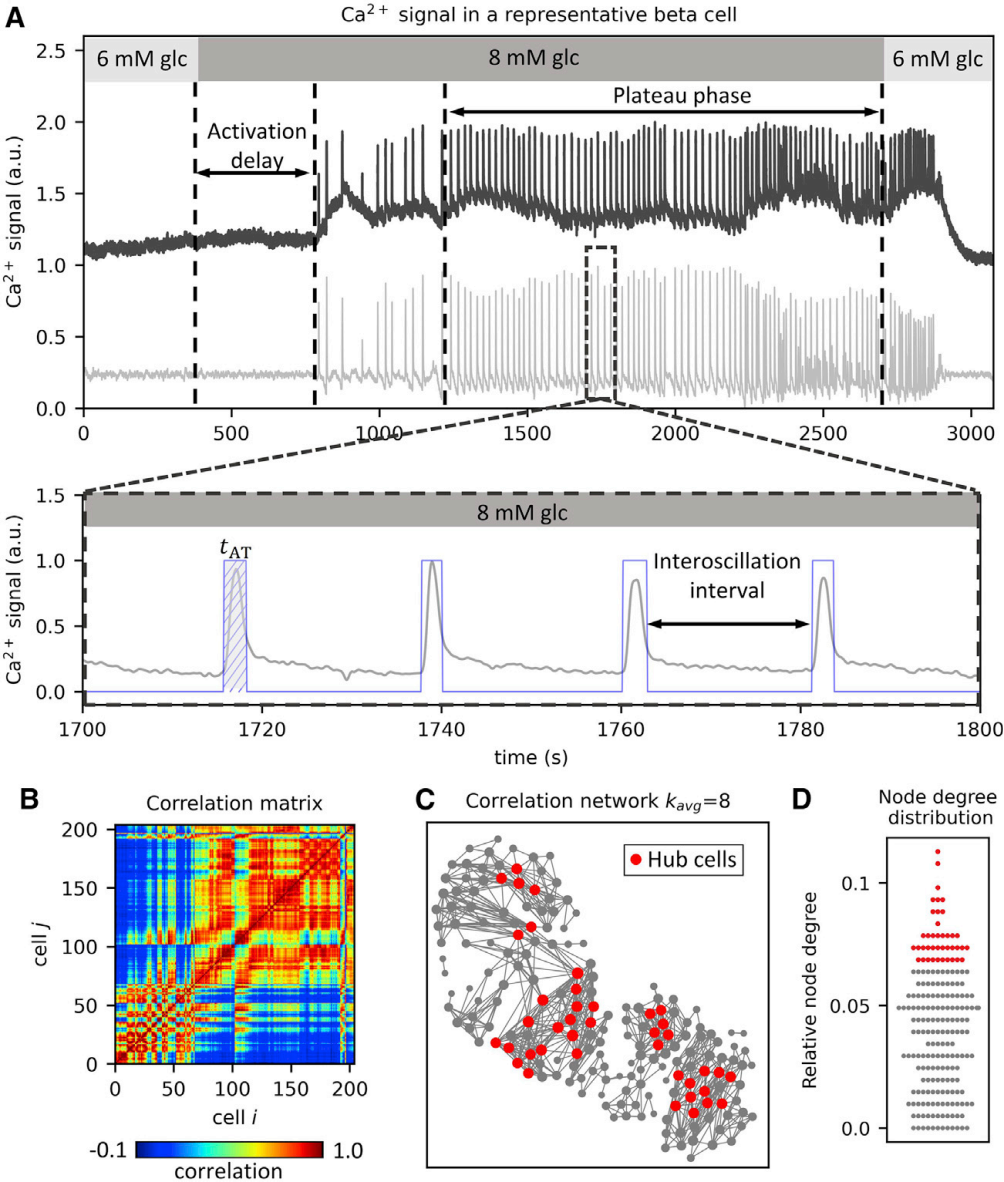
Methodology to analyze Ca^2+^ signals and construct functional correlation-based β cell networks. (*A*) Raw Ca^2+^ signal in a representative β cell (*upper panel*, *dark gray line*) during glucose stimulation with 8 mM glucose. Depicted are the activation delay after switching to stimulatory conditions, and the plateau phase of sustained oscillatory activity. The light gray line below represents the corresponding processed β cell signal. The lower panel shows a zoom-in on the plateau phase of the processed Ca^2+^ signal. The blue line represents the corresponding binarized activity used for further analysis. The hatched area denoted by *t*_AT_ signifies the active time of one selected Ca^2+^ oscillation. (*B*) Correlation matrix computed based on pairwise comparisons of Ca^2+^ signals. (*C*) A representative correlation-based functional β cell network. Nodes represent the physical locations of β cells, and the connections signify cells with highly correlated cellular activity. Red dots indicate the cells with the most connections (i.e., 17% hub cells with the highest node degree values). (*D*) Swarm plot presenting the relative node degree distribution. Red dots indicate the 17% of most connected cells (as in (*C*)). Individual node degrees were normalized with the islet size, i.e., the number of all cells in the islet. To see this figure in color, go online.

Ca^2+^ traces (fluorescence signals of Calbryte 520 AM) for manually selected ROIs were exported as the F/F_0_ ratio employing custom software (ImageFiltering, copyright Denis Špelič). Signals with extensive artifacts or a too low signal-to-noise ratio and those with evident non-β cell-like features were excluded from further analysis. The activation delay of the first phase response, i.e., the delay for initial increase in activity following glucose stimulation (39), was assessed manually for each ROI from unprocessed time series. For the plateau phase activity, i.e., the phase of sustained oscillatory activity following the first phase, all Ca^2+^ traces were processed with a zero-lag band-pass filter (cutoff frequencies 0.05 and 1.0 Hz) and additionally smoothed with an adjacency averaging procedure. After smoothing, traces were binarized (48), and binarized time series were used to calculate the relative active time and the interoscillation interval variability. The former reflects the average fraction of time that cells spend in an active state with increased [Ca^2+^]_i_ and was simply determined as a fraction of 1 (i.e., “on” states), whereas the latter indicates the regularity of oscillations and is defined as the ratio between the SD of interoscillation interval lengths and the corresponding mean interval length (69). Binarized traces were also used to extract individual intercellular Ca^2+^ waves and to determine the activation sequence of β cells within individual waves, as explained in continuation. The methodology of Ca^2+^ signal processing is illustrated in Fig. 1 *A*.

### Assessing hub and wave-initiator cells with network analysis

We first briefly describe the general principles of network analyses that we used to determine hub and wave-initiator cells along with their signaling characteristics. Both subpopulations were in principle determined on the basis of the whole plateau phase of sustained activity (20–60 min intervals). Hub cell populations were determined from functional correlation-based networks from which we extracted 1/6 of the cells with the most functional connections. To determine wave-initiator cells, we constructed network layers for each Ca^2+^ wave and determined the cells that were among the wave initiators—the 1/6 of cells that most often activated among the first within individual waves were deemed as the wave-initiating subpopulation. Typically, a series of 80–120 wave-based network layers was used for this analysis. For the extracted subpopulations we then separately investigated whether they exhibit exceptional Ca^2+^ signaling characteristics and assessed their potential overlap. Specifically, we analyzed how the relative active time, interoscillation interval variability, and activation delay depend on the number of functional connections and on the initiation parameter value. It should be noted that, with the analyses of activation delays, i.e., the time lag of individual cells responding to stimulation, we studied the possible relation to the so-called first responding cell subpopulation. Moreover, we investigated the relationship between functional and wave-based network characteristics with emphasis on the relation between hub and wave-initiator cells. Finally, we also determined the temporal persistency of hub and wave-initiator cells. The latter was assessed by evaluating the role of cells between consecutive wave-based network layers. For the former, we additionally generated evolving functional correlation-based networks. Specifically, we have used rolling correlation analysis to generate a temporal series of functional networks and determined how the number of functional connections and the role of hub cells evolved throughout the whole interval of sustained activity. All methodological details are further explained in the following subsections.

### Construction of correlation-based functional networks

We computed the pairwise Pearson’s correlation coefficients to quantify the collective β cell activity during the whole plateau phase of sustained activity. By this means, correlation matrices (Fig. 1 *B*) were determined for each islet. These were then used to construct functional networks (Fig. 1 *C*) in which nodes represent individual β cells, and their locations correspond to the physical positions of cells in tissue slices (54,55). Connections were established based on the correlation matrix. To avoid an arbitrary threshold selection, we used variable thresholds to extract the networks so that the average node degree (number of connections per cell) in all islets was $k_{avg}\approx 8$. This seemingly arbitrary number was used to mimic realistic β cell connectivity in the tissue (70). However, it should be noted that within reasonable limits the results are qualitatively independent of the exact value (62), as we also demonstrate in our supporting material. Heterogeneity in network connectivity was assessed through the computation of node degrees. Fig. 1 *D* shows the distribution of node degrees for a representative islet with indicated 1/6 (17%) of the most connected cells, i.e., hub cells.

### Characterizing intercellular signals with Ca^2+^ wave-based network layers

**Figure 2:**
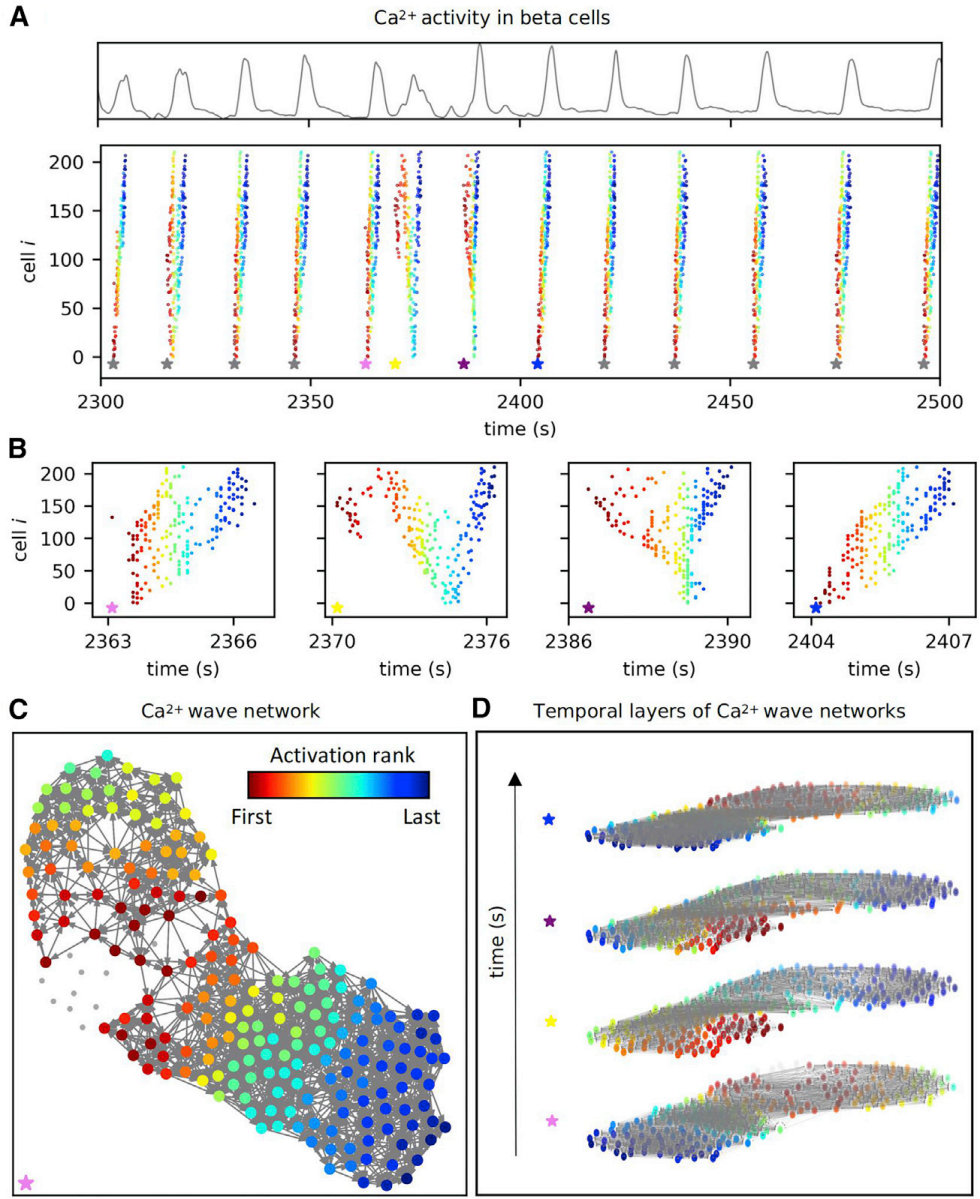
Intercellular Ca^2+^ wave analysis. (*A*) Average Ca^2+^ signal of a representative islet (*upper panel*) and corresponding raster plot with indicated oscillation onsets in individual intercellular Ca^2+^ waves (*lower panel*). The colors of dots represent the activation rank of individual cells within each wave, with red and blue colors denoting the first and last activated cell, respectively (see also the color bar in (*C*). Stars indicate the time point of the onsets of different Ca^2+^ waves. (*B*) Raster plots for four subsequent Ca^2+^ waves (see the color of the stars to link with the data on (*A*)). (*C*) Directed Ca^2+^ wave network extracted from the first Ca^2+^ wave in (*B*) in which the colors of the cells reflect the sequence of activation. The activation ranks are color coded as indicated by the color bar. (*D*) The sequence of the four successive Ca^2+^ waves is presented as a multilayer temporal network. The connections and the colors of cells have the same meaning as in (*C*). To see this figure in color, go online.

Individual intercellular Ca^2+^ waves were extracted from binarized cellular signals using a space-time clustering algorithm described before (48). In short, cells *i* and *j* were considered part of the same wave, i.e., wave-network layer *α*, if their onsets of oscillations (*T*_*i*_ and *T*_*j*_) were close enough in space and time. Specifically, the spatial threshold *R*_s_ was determined on the basis of the average intercellular distance and the corresponding SD: $R_{s}=<d_{ij}>-xSD\left(d_{ij}\right)$, where *d*_ij_ is the distance between cells *i* and *j* in a given islet. For the temporal threshold we took *R*_*t*_ = 0.7 s, which roughly corresponds to 1/3 of the total time for a wave to travel across the whole islet. Most importantly, within reasonable and physiologically meaningful values of the threshold, our analyses are qualitatively independent on the choice of their values, as we also demonstrate in the supporting material. Moreover, all cells belonging to the given Ca^2+^ wave were ranked by their order of activation (Fig. 2 *A*). Typically, between 80 and 120 waves were detected during the plateau phase per islet, and the wave front encompassed cells spanning over several tens of micrometers, implying that relatively small errors in binarization due to inherent experimental noise in the Ca^2+^ signal could result in inconsistent coordinates of wave initiators. In an attempt to overcome this issue, each β cell was assigned an initiation parameter. The initiation parameter was defined as the fraction of waves in which a cell was within the smallest 10% of activation ranks. The distribution of the initiation parameter per islet was used to define cells with the highest tendency to initiate waves (i.e., wave initiators) using an arbitrary cutoff value of 17% (1/6). Furthermore, weighted and directed connections between all cell pairs *i* and *j* within each wave-based layer *α* were established, and the direction was *i* − > *j* if *T*_*i*_ < *T*_*j*_ and vice versa. If the two cells activated simultaneously, they were connected with an undirected connection (Fig. 2 *C*). Weights of the connections were determined based on the time delays between the activations of cells *i* and *j* within the wave ($\left|T_{i}-T_{j}\right|$). Therefore, the weights reflect how fast the excitation signal traveled between different cell pairs. The average weights of individual cells determined from all temporal layers were used as a proxy for intercellular coupling, as they encode the information on how fast on average a given cell transmits intercellular waves. As demonstrated previously, the efficiency of intercellular coupling between direct neighbors depends on the gap junctional conductivity and the so-called input conductance, which is essentially the nonjunctional plasma membrane conductance, with the K_ATP_ and Ca^2+^-dependent potassium conductance playing major roles. To complicate things further, both junctional and membrane conductance change or may change repeatedly during the plateau phase of fast oscillations. We elaborate this into more detail in the discussion. By this means, each wave-based network layer contains a set of connections encompassing the course of the wave and the transmission delays along its path. Our analysis considered only intercellular Ca^2+^ waves in which more than 45% of all cells in the islet participated. To assess the interwave similarity and the persistency of wave initiators, we constructed multilayer networks by stacking all waves on top of each other in chronological order for each recording (Fig. 2 *D*) (48), as explained in more detail in continuation. Since in this work we, for the first time, use two different kinds of networks, it is worth explicitly pointing out that they are fundamentally different. The correlation-based network (Fig. 1) is the now standard approach to constructing and analyzing functional network properties, such as the number of links, based on similarities between long Ca^2+^ traces that each contain a large number of fast oscillations, whereas the Ca^2+^ wave-based network (Fig. 2) is a new approach to quantify intercellular wave propagation by means of directed weighted graphs and each such graph is essentially based on a single intercellular wave that synchronizes a single fast oscillation, and tens of such graphs are then pooled to obtain wave network parameters, such as the initiation parameter.

### Quantifying the overlap of β cell subpopulations

We quantified the extent of overlap between the four subpopulations of cells: 1) first responders (during the first phase), 2) initiators of waves (i.e., wave-initiator cells), 3) most active cells (during the plateau phase), and hub cells (cells with a high number of functional connections during the plateau phase). The subpopulations of hub and wave-initiator cells were defined above. First responder cells were defined as the top 17% (1/6) of the cells that responded first to stimulatory glucose concentrations (8 or 12 mM). The most active cells were defined as the top 17% (1/6) of the cells with the highest relative active time values. We computed the pairwise overlap of cellular subpopulations ($O_{i,j}$) as the ratio of the actual overlap probability ($P_{overlap}^{i,j}$) and the probability that cells belong to both subpopulation *i* and *j* by chance ($P_{rand}$):

(1) $$O_{i,j}=\frac{P_{overlap}^{i,j}}{P_{rand}}\;i,j\in \left[Hubs,Initiators,First\;responders,Most\;active\right]\;i\neq j\text{,}$$

where $P_{overlap}^{i,j}=\frac{m\left(i\cap j\right)}{N}$; m(i∩j) is the cardinality of the overlap of subpopulations *i* and *j* and *N* is the number of cells in the recording, and $P_{rand}=\frac{m\left(i\right)m\left(j\right)}{N^{2}}$ (m(i) and m(j) are the cardinalities of sets *i* and *j*).

### Temporal persistency of the initiator and hub cells

For each detected Ca^2+^ wave *α*, a set of initiator cells ($I^{\alpha }$) was constructed based on the aforementioned 10% of the first activated cells within individual Ca^2+^ waves. Temporal initiator cell stability *IS* was calculated as the relative overlap of the sets of initiator cells in waves α and $\alpha^{\prime }$ as the so-called Jaccard similarity:

(2) $${IS}_{\alpha ,\alpha^{\prime }}=\frac{I_{shared}^{\alpha ,\alpha^{\prime }}}{I_{unique}^{\alpha ,\alpha^{\prime }}}\text{,}$$

where $I_{shared}^{\alpha ,\alpha^{\prime }}$ is the cardinality of the intersection of the sets of initiator cells and $I_{unique}^{\alpha ,\alpha^{\prime }}$ is the cardinality of the union of the two sets of initiator cells. Equation (2) yields a value between 0 and 1, where 0 means there are no cells in the intersection and 1 means a perfect intersection. The latter reflects the scenario where all initiator cells in layers α and α′ were the same. With Eq. (2), we constructed initiator similarity matrices for all Ca^2+^ wave pairs α and $\alpha^{\prime }$ in individual recordings and calculated the average initiator similarity $\bar{IS}\left(n\right)$ as a function of the temporal distance for all wave pairs that were *m* steps apart as:

(3) $$\bar{IS}\left(m\right)=\frac{1}{M}\sum_{\alpha =1}^{M}IS_{\alpha ,\left(\alpha +m\right)}\text{,}$$

where m=1,2,… and *M* is the number of Ca^2+^ waves that are *m* steps apart.

To assess the temporal persistency of functional connectivity patterns and hub cells, we computed the temporal evolution of the correlation-based network using a sliding window of $T_{sw}=300$ s. By this means we generated a temporal series of functional networks rolling over the whole plateau phase, so that networks generated in each step represent a temporal layer β with $k_{avg}\approx 8$. To ensure comparison with the analysis of the initiator cell persistency, we used a temporal step size equal to the average interoscillation interval in each recording. In this case, the temporal distance between two layers β and βʹ roughly corresponds to the interval between two subsequent Ca^2+^ waves α and $\alpha^{\prime }$, which are, on average, also separated by the same temporal distance. The average node degree in all temporal layers and the corresponding SDs were calculated for each cell in the islet. Moreover, in each layer β, 17% (1/6) of the cells with highest node degrees were designated hub cells and formed a set $H^{\beta }$. Temporal hub similarity (71) was then assessed via the Jaccard similarity index as the relative overlap of the sets of hub cells in temporal layers β and $\beta^{\prime }$ as:

(4) $${HS}_{\beta ,\beta^{\prime }}=\frac{H_{shared}^{\beta ,\beta^{\prime }}}{H_{unique}^{\beta ,\beta^{\prime }}}\text{,}$$

where $H_{shared}^{\beta ,\beta^{\prime }}$ is the cardinality of the intersection of the sets of hub cells, and $H_{unique}^{\beta ,\beta^{\prime }}$ is the cardinality of the union of the two sets of hub cells. This yields a value of *HS* for layers β and $\beta^{\prime }$ between 0 (empty intersection) and 1 (perfect intersection), with the latter representing the scenario where hub cells in both layers *β* and *β*′ were the same. With Eq. (4), we constructed hub similarity matrices for all layer pairs β and $\beta^{\prime }$ in individual recordings and calculated the average hub similarity $\bar{HS}\left(n\right)$ as a function of temporal distance for all pairs that were *n* layers apart as:

(5) $$\bar{HS}\left(n\right)=\frac{1}{N}\sum_{\beta =1}^{N}HS_{\beta ,\left(\beta +n\right)}\text{,}$$

where n=1,2,… and *N* is the number of all network layer pairs that are *n* layers apart.

### Data pooling, normalization, and statistical analysis

To compare different signaling parameters from all cells in different islets, we first normalized all values with the average value of the parameter in the islet, yielding values distributed around unity. We pooled the data separately for 8 and 12 mM glucose stimulation. Next, pooled parameter values were separated into the lowest 1/6, intermediate 2/3, and highest 1/6 tier. Statistical significance of differences (p values) between individual tiers for each parameter was calculated with the Kruskal-Wallis one-way analysis of variance on ranks with a post hoc pairwise multiple comparison procedure (Dunn’s method). In addition to p values, we also calculated the effect size δ (Cliff’s Delta) of differences between pairs of parameter tiers $Q_{i}$ and $Q_{j}$ (i,j ∈ [lowest 1/6, intermediate 2/3, and highest 1/6], i≠j) as follows (72):

(6) $$\delta =\frac{{m(Q}_{i}>Q_{j})-m(Q_{i}<Q_{j})}{m\left(Q_{i}\right)\;m\left(Q_{j}\right)}\text{,}$$

where ${m(Q}_{j}>Q_{j})$ and $m\left(Q_{i}<Q_{j}\right)$ are the cardinalities of the subsets, where $Q_{i}$ is larger than $Q_{j}$ and vice versa, and ${m(Q}_{i})$ and ${m(Q}_{j})$ are the cardinalities of the two tiers of the parameter. The effect size was then categorized as negligible – N (δ < 0.147), small – S (0.33 > δ ≥ 0.147), medium – M (0.474 > δ ≥ 0.33), or large – L (δ ≥ 0.474) depending on the value of δ (73).

## Results

We applied functional multicellular calcium imaging and in-depth network analysis to assess β cell roles and functional parameters within the pancreatic islets. We created functional correlation-based networks to identify highly interconnected cells that act as signal transduction hubs (i.e., hub cells). We also created intercellular Ca^2+^ wave-based networks to identify Ca^2+^ wave-initiator cells and describe the course of the intercellular signals. Recordings of β cell activity in acute tissue slices were performed either under physiological (8 mM) or supraphysiological (12 mM) glucose concentration.

### Analyzing the Ca^2+^ signaling characteristics of hub and wave-initiator cells

**Figure 3:**
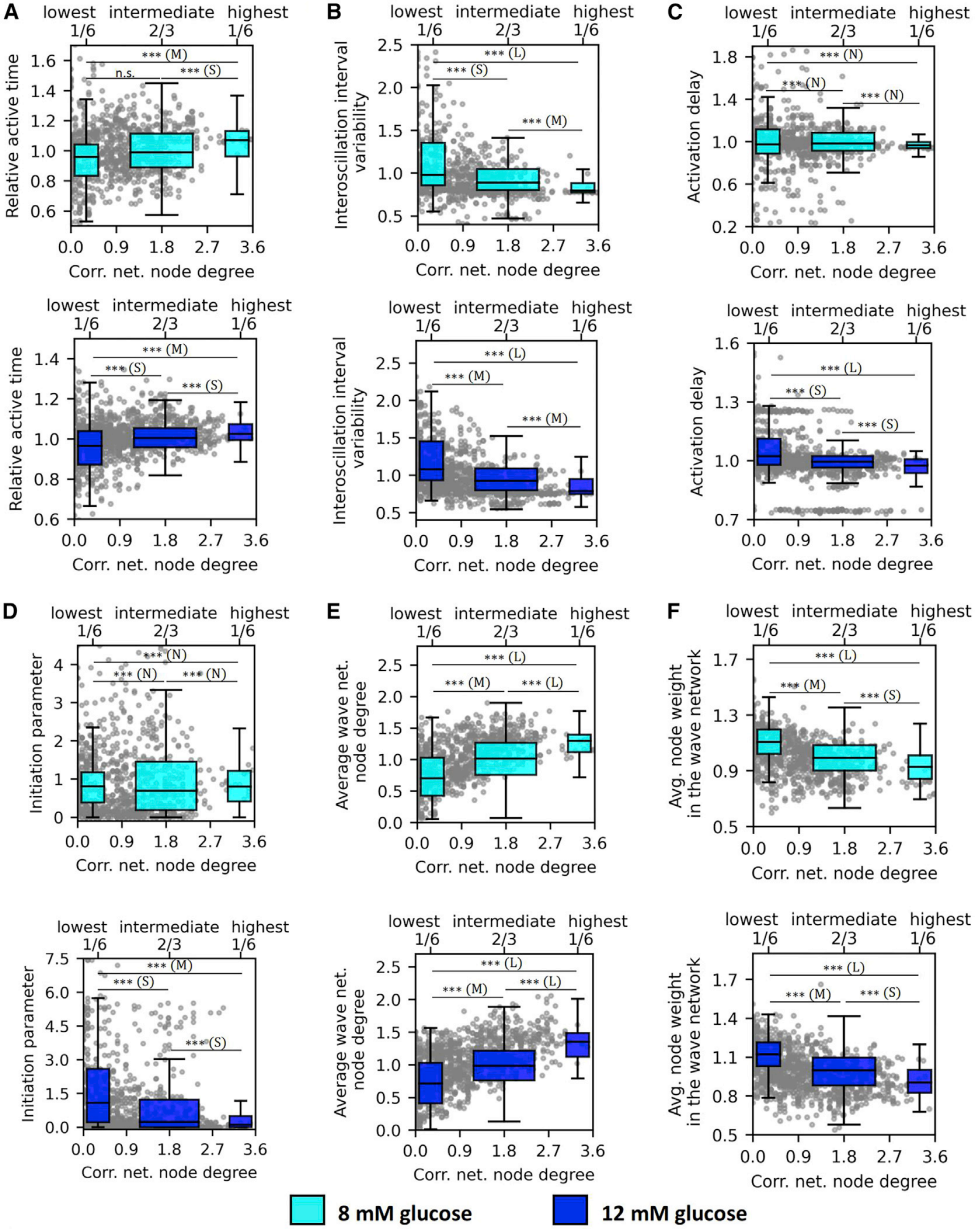
Relationship between various Ca^2+^ signaling parameters and the number of functional connections in correlation-based networks. Dependence of the relative active time (*A*), interoscillation interval variability (*B*), activation delay (*C*), initiation parameter (*D*), wave-network node degree (*E*), and the average node weight in the wave network, i.e., average transmission delay (*F*) on the correlation network degree for stimulation protocols with 8 mM glucose (*first and third row*, *cyan*) and 12 mM glucose (*second and fourth row*, *blue*). Gray dots represent normalized values of individual cells, and boxplots show the lowest 1/6, the middle 2/3, and the highest 1/6 connected cells in the functional network. All panels show the pooled data from all recordings whereby individual values were normalized by the average value of the specific parameter in the given islet. Boxes determine the interval between the 25th and the 75th percentile, whiskers denote the 10th and the 90th percentile, and lines within the boxes indicate the median. Data were pooled from the following number of islets/cells: 8/865 (8 mM glc); 8/1103 (12 mM glc). Statistical significance: ^∗^p < 0.05, ^∗∗^p < 0.01, ^∗∗∗^p < 0.001; n.s., not significant. Effect sizes: negligible – N (δ < 0.147), small – S (0.33 > δ ≥ 0.147), medium – M (0.474 > δ ≥ 0.33), or large – L (δ ≥ 0.474). To see this figure in color, go online.

First, we investigated how cellular signaling parameters relate to their corresponding node degree, i.e., the number of functional connections in the correlation-based network. Results are shown in Fig. 3 for all recordings performed with 8 mM (*upper panels*, *cyan*) and 12 mM (*lower panels*, *blue*) glucose stimulation. Data points for individual cells (*gray dots*) are shown in each panel, along with the collective data for cells with the 1/6 lowest, 2/3 intermediate, and 1/6 highest correlation network node degrees (boxplots). Note that the signaling parameter values of each cell were normalized with the average value of their corresponding islet to allow the comparison between different islets. Fig. 3 *A* shows the relative active time of cells as a function of the node degree. A clear correlation between the number of functional connections and the relative active time was observed in both glucose concentrations, indicating that the hub cells tend to be among the most active cells.

In contrast, a stark anticorrelation was found between the interoscillation interval variability and the node degrees (Fig. 3 *B*). The cells with the most functional connections exhibited the most regular calcium oscillations, and the trend was prominent under both stimulatory glucose levels. In Fig. 3 *C*, we show how the delay in the onset of Ca^2+^ oscillations after switching from substimulatory to stimulatory glucose depends on the node degree. There appears to be no correlation between activation delays and node degrees under 8 mM glucose, while under 12 mM glucose the most connected cells tended to activate first.

Next, we examined the interrelations between cells' roles in the correlation-based functional networks and in the Ca^2+^ wave-derived network layers. Fig. 3 *D* shows the relation between the initiation parameter values of individual cells and the number of functional connections. It turned out that there was no discernible correlation under physiological stimulation, whereas under supraphysiological stimulation, a slight anticorrelation was inferred. These results indicate that the most connected cells in the correlation-based functional networks, i.e., hub cells, do not principally act as wave initiators in the wave-based networks of the same islets and that, under supraphysiological conditions, the waves are actually more likely to be initiated from less connected cells. Moreover, there is a powerful relation between the number of connections each cell has in the functional and Ca^2+^ wave-based network (Fig. 3 *E*), which substantiates their central role in transmitting intercellular waves. Most importantly, results in Fig. 3 *F* reveal a compelling correlation between the node degree in the correlation network and the average node weights in the wave-based network. The latter reflects the delays in intercellular signal transmission, which implies that hub cells exhibit a stronger intercellular coupling when compared with cells with less functional connections. It should be noted that the average node degree in the wave-based network (Fig. 3 *E*) portrays only the number of connections with direct neighbors in the observed focal plane, without taking into account their weights, whereas the average node weight (Fig. 3 *F*) is computed as the sum of all in- and out-weights of connections divided by the number of connections of this cell. In other words, hub cells, as defined by the correlation-based network, have both a higher number of direct neighbors in the wave-based network and the average phase-shift between their own oscillations and oscillations in their neighbors are significantly shorter. Finally, since theory predicts that the velocity of waves should not change with increasing glucose (see (86) and discussion for more details) we quantified the glucose dependence of node weights in the wave-based network (Fig. S1). The range of average node weights was 0.1–0.35 s and thus consistent with a wave velocity in the range of 30–100 *μ*m/s. In hub cells, the average node weight was approximately 20% lower in both glucose concentrations. Most importantly the node weight, corresponding to the temporal delay between neighboring cells, did not importantly differ between 8 and 12 mM glucose.

**Figure 4:**
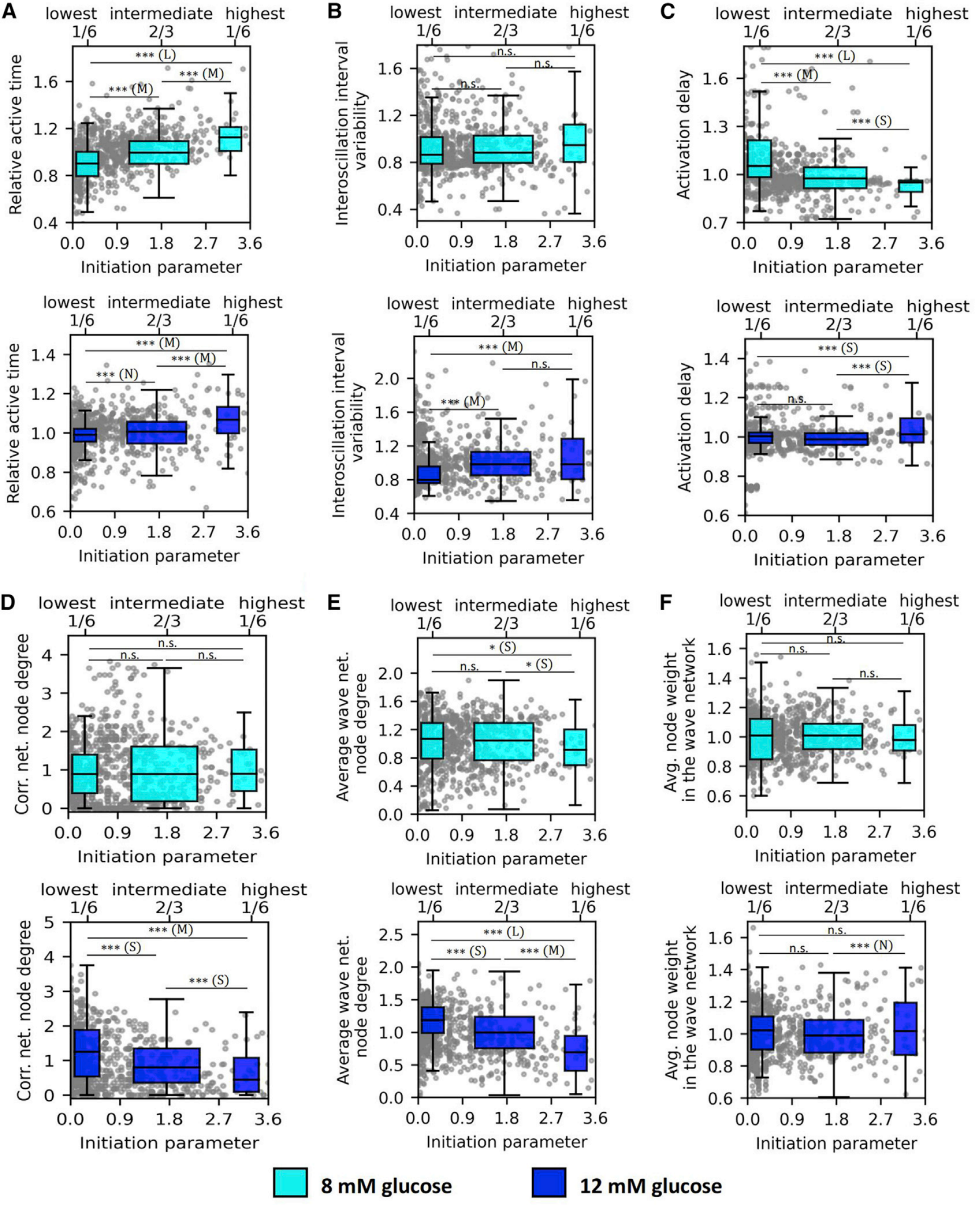
Relationship between various Ca^2+^ signaling parameters and the wave initiation parameter. Dependence of the relative active time (*A*), interoscillation interval variability (*B*), activation delay (*C*), correlation-based network node degree (*D*), wave-network node degree (*E*), and average node weight in the wave network (*F*) on the initiation parameter for stimulation protocols with 8 mM glucose (*first and third row*, *cyan*) and 12 mM glucose (*second and fourth row*, *blue*). Gray dots represent values of individual cells, and boxplots show the lowest 1/6, the intermediate 2/3, and the highest 1/6 connected cells in the correlation network. All panels show the pooled data from all recordings whereby individual values were normalized by the average value of the specific parameter in the given islet. Box charts are defined as in Fig. 3. Boxes determine the interval between the 25th and the 75th percentile, whiskers denote the 10th and the 90th percentile, and lines within the boxes indicate the median. Data were pooled from the following number of islets/cells: 8/865 (8 mM glc); 8/1103 (12 mM glc). Statistical significance: ^∗^p < 0.05, ^∗∗^p < 0.01, ^∗∗∗^p < 0.001; n.s., not significant. Effect sizes: negligible – N (δ < 0.147), small – S (0.33 > δ ≥ 0.147), medium – M (0.474 > δ ≥ 0.33), or large – L (δ ≥ 0.474). To see this figure in color, go online.

We then examined how different cellular signaling parameters depend on the Ca^2+^ wave initiation parameter to characterize wave-initiator cells. Results are shown in Fig. 4, where panels in Fig. 4 *A* clearly demonstrate that the wave-initiating cells are the most active under physiological as well as under supraphysiological stimulation levels. Interestingly, in contrast to hub cells (Fig. 3 *B*), these wave-initiator cells are not exceptional in terms of the regularity of oscillations (Fig. 4 *B*). Fig. 4 *C* presents the activation delays of cells after the onset of stimulation depending on the initiation parameter. Upon stimulation with 8 mM glucose, there was a tendency of the wave initiators responding first during the first phase, whereas under 12 mM glucose, no correlation between these parameters was inferred.

Furthermore, our results indicate that the wave initiation parameter relates very weakly to the cells role in the β cell networks. In 8 mM glucose, there was no correlation, whereas, in 12 mM glucose, wave-initiator cells tended to have a lower-than-average number of connections in the correlation-based network (Fig. 4 *D*). Interestingly, in 8 mM and especially in 12 mM glucose, the wave initiators tended to have a lower-than-average number of links with their neighbors in the wave-based network. Moreover, in neither of the two glucose concentrations, a correlation between the initiation parameter and the average intercellular signal transmission delay between neighboring cells was observed, i.e., average weighted wave-based network node degree (Fig. 4 *F*). The above indicates that the cells that often serve as wave initiators are not distinguished by their strength of intercellular coupling with respect to individual neighbors, however, they tend to have fewer neighbors.

To design functional and wave-based networks, to determine hubs and wave initiators, and to categorize cells to groups, we have used a specific set of parameters, which were determined based on the nature of the data as well as physiological relevance (see materials and methods). However, for the sake of scientific rigor, we additionally investigated whether our findings are sensitive to the choice of these parameters. In the supplementary figures we therefore present the calculations obtained for a broad range of different values of these parameters. We first tested whether the arrangement of cells affects the conclusions and performed two additional data splits: 1) lowest 1/10, intermediate 4/5, highest 1/10 cells (Figs. S2 and S3), and 2) lowest 1/4, intermediate 1/2, highest 1/4 cells (Figs. S4 and S5) for the correlation network node degree and for the initiation parameter. Results clearly show that all investigated signaling parameters follow the same trend regardless of the selected data split. Next, we investigated the impact of the selected average correlation network node degree on the identified hub cells and the most notable signaling parameters (relative active time, interoscillation interval variability, wave network node weight, and initiation parameter). We performed the analysis on a representative islet for average correlation network node degrees *k*_avg_ = 6.0, *k*_avg_ = 8.0, and, *k*_avg_ = 10.0 (Fig. S6). The identified hub cells (1/6 of most connected cells) are largely independent of the selected average node degree (Fig. S6 *A*) and the selected signaling parameters also appear to follow the same trend for each value (Fig. S6 *B*). Finally, we analyzed whether the wave-network layer extraction is sensitive to the distance and delay threshold parameters for wave detection, i.e., *R*_s_ and *R*_t_ (see materials and methods). To that end, we performed the same wave detection process for multiple combinations of those parameters on a representative islets plateau phase and present the findings in Fig. S7. There is only a minor difference in the number of detected waves, which indicates that within reasonable limits our results are not sensitive to the specific values of these parameters.

**Figure 5:**
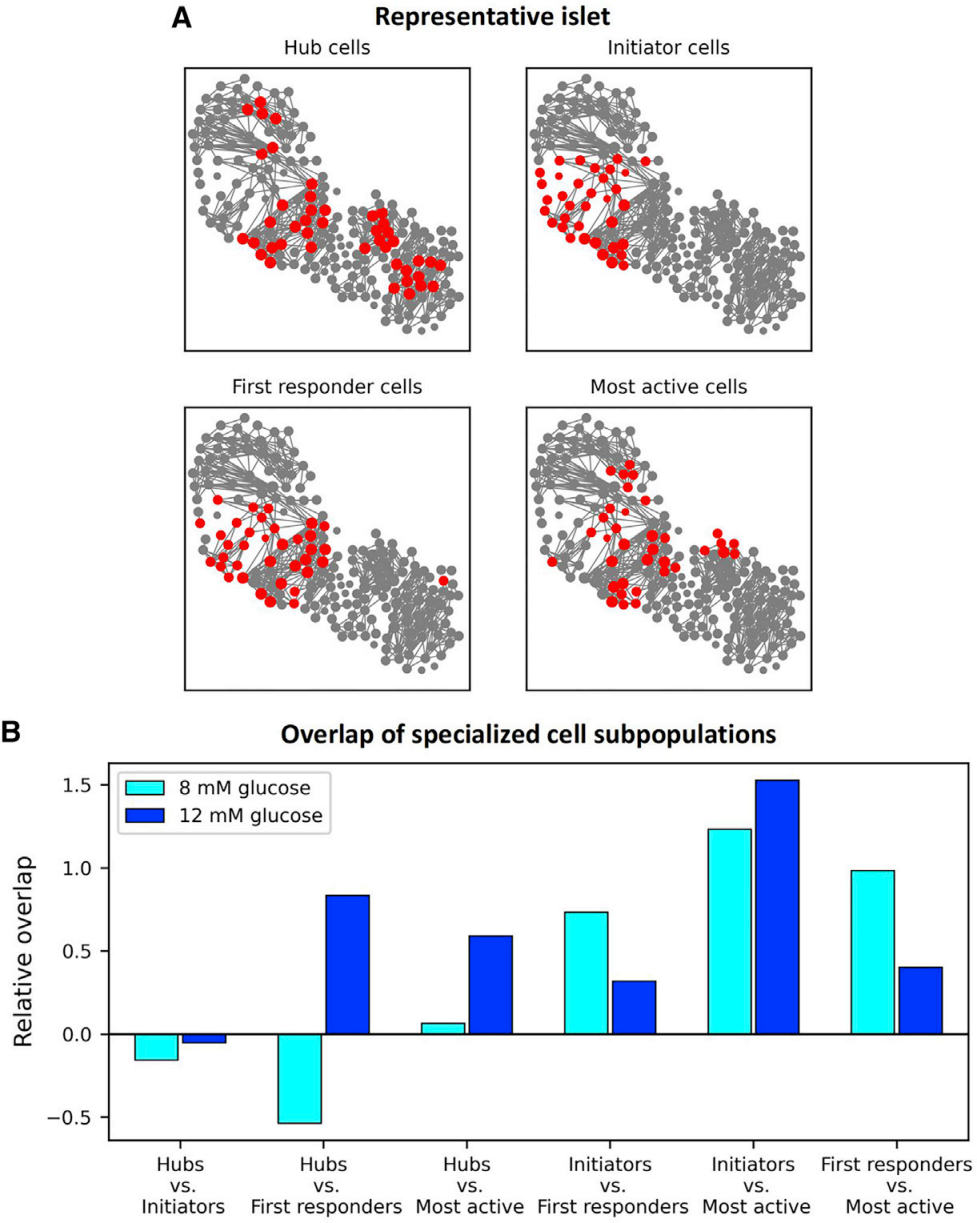
Characterizing the overlap of specific β cell subpopulations. (*A*) Correlation-based functional β cell network extracted from a typical islet. Node size reflects the number of functional connections per cell. The red-colored cells indicate 1/6 of the 1) most connected cells, i.e., hub cells (*upper left*), 2) wave initiators, i.e., cells with the highest initiation parameter values (*upper right*), 3) first responding cells to stimulatory glucose concentrations, i.e., cells with the lowest activation delays (*lower left*), and 4) most active cells, i.e., cells with the highest active times (*lower right*). (*B*) Bar plots show the relative overlap of specific cell subpopulations compared with the overlap by chance that two cells belong to the same sets of subpopulations (zero means an approximately 2.8%, i.e., (1/6)^2^, overlap). To see this figure in color, go online.

In the following, we investigated whether there is an overlap between specific β cell subpopulations. The results in Fig. 5 *A* feature a typical islet with marked top 1/6 of cells with the most functional connections (hubs), cells with the highest initiator parameter values (wave initiators), cells that responded first to stimulation (first responders), and that had the highest relative active time (most active cells). A visual inspection of the plot indicates some overlap between specific cell subpopulations. To investigate this further, we quantified the overlap of cells in all recordings in 8 mM (*cyan bars*) and in 12 mM (*blue bars*) glucose (Fig. 5 *B*). The relative overlaps of specific cell subpopulations are expressed concerning the overlap by chance, such that 0 corresponds to overlap purely by chance (see the materials and methods section for further details). The results reveal that the intersections between the designated cell subpopulations are not large. In both glucose concentrations, the overlap is rather well pronounced only between the wave-initiating and the most active cells, where approximately 42% of specific cell types were present in both groups. There also appears to be a certain overlap between the initiators and the first responders and between the first responders and the most active cells, but only under physiological stimulatory conditions. The relation between the hub and the first responding cells seems to be very glucose dependent, as in 8 mM a negative overlap was detected. In contrast, in 12 mM glucose, the opposite was observed. Finally, practically no overlap between the hub and the wave-initiator cells was detected in either glucose concentration, indicating that, in principle, they represent different and independent β cell subpopulations.

### Assessing the temporal persistency of hub and wave-initiator cells

**Figure 6:**
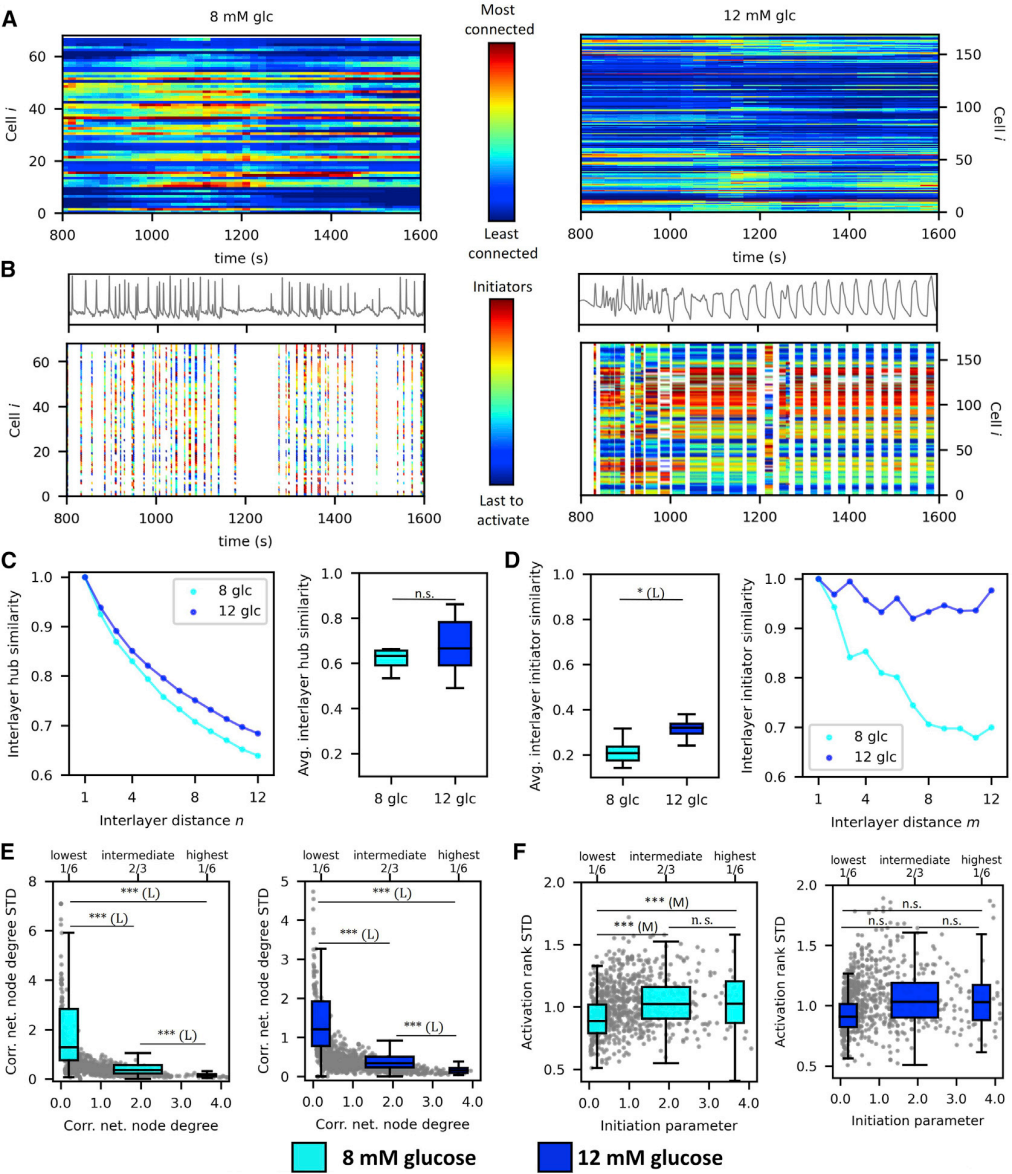
Assessing the temporal persistency of hub and wave-initiator cells. (*A*) The evolution of relative node degrees in the functional β cell network within a sliding temporal window (width 300 s, step 20 [8 mM] and 5 [12 mM] s) in two typical islets with 8 mM (*left*) and 12 mM (*right*) glucose stimulation. The relative number of functional connections is color coded as indicated by the color bar. (*B*) Upper panels show the mean-field Ca^2+^ signal of the same two representative islets as in (*A*), and lower panels show raster plots indicating individual Ca^2+^ oscillations with color-coded activation sequence within individual Ca^2+^ waves. The ranks for oscillation onsets are color coded as indicated by the color bar, such that the red dots reflect the initiating cells within a given wave. (*C*) Average interlayer hub similarity as a function of interlayer distance *n* (*left*) and the average values of interlayer hub similarity (*right*). (*D*) The average values of wave-network interlayer initiator similarities (*left*) and the average wave-network interlayer initiator similarity as a function of the interlayer distance *m* (*right*). (*E*) Temporal variability of node degrees in correlation-based networks dependent on the node degree. Gray dots denote the values of individual cells, and the boxplots the 1/6 lowest, 2/3 intermediate, and 1/6 highest connected cells. (*F*) Temporal cellular activation rank variability dependent on the initiation parameter. Gray dots represent the values of individual cells, and the boxplots the 1/6 lowest, 2/3 intermediate, and 1/6 highest connected cells. Boxes determine the interval between the 25th and the 75th percentile, whiskers denote the 10th and the 90th percentile, and lines within the boxes indicate the median. (*C–F*) The pooled data from the following number of islets/cells: 8/865 (8 mM glc); 8/1103 (12 mM glc). Statistical significance: ^∗^p < 0.05, ^∗∗^p < 0.01, ^∗∗∗^p < 0.001; n.s., not significant. Effect sizes: negligible – N (δ < 0.147), small – S (0.33 > δ ≥ 0.147), medium – M (0.474 > δ ≥ 0.33), or large – L (δ ≥ 0.474). To see this figure in color, go online.

Finally, we analyzed the temporal persistence of hub and wave-initiator cells over the course of the plateau phase of a single square pulse stimulation by glucose. In Fig. 6 *A*, we present color-coded temporal evolution of node degrees for each cell for two representative islets stimulated with 8 mM (*left*) and 12 mM (*right*) glucose. In each time step, node degrees were computed from the correlation-based networks within a sliding window of *T*_sw_ = 300 s, and the values were normalized relative to the highest node degree. It appears that there are some variations in node degrees during 8 and 12 mM glucose, but in general, the roles of the hub and nonhub cells remained preserved even after prolonged exposure to glucose. To assess the temporal stability of wave-initiator cells, we visualized the cellular activation sequences within individual Ca^2+^ waves as raster plots with indicated Ca^2+^ oscillations (Fig. 6 *B*). The colors of stripes denote cellular activation ranks, with the red color denoting the cells that activated first during a given wave, as indicated by the color bar. The plots indicate that, in 8 mM glucose, the activation patterns are rather changeable, whereas, in 12 mM, the activation patterns seem to be relatively stable even over prolonged periods. To obtain a general insight and to quantify how stable hubs, wave-initiator cells, and the paths of Ca^2+^ waves are across time, we computed the interlayer similarity between correlation- and wave-based temporal network layers (see materials and methods for details) for all recordings in 8 and 12 mM glucose. In Fig. 6 *C*, we show the average absolute values of the hub interlayer similarity (*right panel*) and the normalized interlayer hub similarity as a function of temporal distance between network layers (*left panel*). Similarly, in Fig. 6 *D*, we show the average absolute values of the interlayer wave initiator similarity (*left panel*) and the normalized interlayer initiator similarity as a function of temporal distance between network layers (*right panel*). It turned out that, on average, the hub similarity between network layers is much higher than the wave initiator similarity, which indicates that the role of hub cells is significantly more persistent than the role of wave-initiator cells.

Moreover, the temporal stability of hub cells did not depend on glucose concentration, whereas the role of wave-initiator cells was found to be more stable in 12 mM compared with 8 mM glucose. The results showing how the normalized values of interlayer similarities decay with the temporal distance further corroborate this notion, as the interlayer Ca^2+^ wave similarity declines much slower in 12 mM than in 8 mM glucose. To further evaluate the characteristics of β cells concerning their roles in correlation- and Ca^2+^ wave-based networks in detail, we quantified the dispersion of temporal node degrees and initiation parameter values. The relationship between the relative correlation-based network node degrees of cells and their corresponding SD shown in Fig. 6 *E* indicates that the number of functional connections fluctuates in time much less in cells with the most functional connections in both glucose concentrations. In contrast, we found no clear relation between the SD of average activation ranks inferred from onsets of Ca^2+^ oscillations within individual waves and the initiation parameter (Fig. 6 *F*). These results suggest that the roles of wave-initiating cells are more variable and less determined than those of the hub cells.

## Discussion

Pancreatic islets are highly interconnected structures and display a fascinating coordination of their rhythmic activity, which plays a key role in the regulation of metabolic homeostasis and becomes progressively impaired in diabetes mellitus. The increasing awareness that the intercellular coupling and its modulation are vital to the normal islet function has stimulated immense interest in how different subpopulations of heterogeneous cells are functionally arranged throughout the islets and how they mediate intercellular signals. Recent advances in optogenetics, photopharmacology, and computational tools have allowed the assessment of multicellular β cell behavior revealing that the mediating Ca^2+^ waves are initiated from specific subregions of the islet with specific local excitability and metabolic profiles (7,25,35,48,59,64,65,74,75,76,77,78). Moreover, network analyses have emerged as promising tools to elucidate the collective activity of β cell populations. It turned out that the functional β cell connectivity patterns are much more heterogeneous than one would expect from a gap junction coupled syncytium, displaying small-worldness and a hub-like connectivity architecture (7,11,42,43,46,49,54,59,61,79). The complexity of these functional interactions results from the intricate islet-wide Ca^2+^ dynamics that is influenced by a multilayered cellular heterogeneity along with heterogeneous intercellular interactions and by the extracellular environment. However, how specific specialized subpopulations of β cells contribute to synchronized dynamics, network activity, persistency, and initiation of intercellular signals and what are their functional characteristics, is not clear and is a matter of ongoing research. Particularly the term pacemaker has in the recent literature been loosely applied to refer to hub cells, wave-initiator cells, or to the first responding cells that control Ca^2+^ elevations in response to stimulatory glucose, as has been clarified in a very recent review by Benninger and Kravets (7). Therefore, classifying specific β cell types and subpopulations, assessing their functional characteristics, and elucidating how the multicellular consortium coordinates the intercellular Ca^2+^ activity and insulin secretion from an islet, have attracted a lot of attention by the islet community and represent very vibrant topics.

In this study, we systematically addressed the aforementioned issues by combining multicellular Ca^2+^ imaging in mouse pancreas tissue slices with network science approaches. We constructed correlation-based functional multicellular networks based on the temporal similarity of the measured cellular dynamics, such that nodes represented individual β cells and connections between them were established based on thresholded pairwise correlations of Ca^2+^ imaging signals (55). By these means, we detected a fraction of very well functionally connected cells, i.e., hub cells. This subpopulation has already been identified before in various experimental (44,46,49,79) and theoretical (37,56,63,80) studies and its existence was suggested to be important for normal islet functioning (7,42,46). Moreover, utilization of advanced optogenetic and photopharmacological strategies has indicated that the hub cells exhibit hyperpolarized mitochondria, a lower insulin content resembling a transcriptionally immature phenotype due to the low expression levels of signature β cell transcription factors, and that they are metabolically highly active (35,46,81). Increased metabolic activity could at least in part be associated with our findings that hub cells exhibit a higher-than-average activity (Fig. 3 *A*), although a clear overlap between hub cells and the most active cells was observed only under 12 mM glucose stimulation and not under 8 mM (Fig. 5 *B*). Moreover, we noticed a tendency of hub cells to exhibit the most regular oscillations (Fig. 3 *B*). This observation indicates that hub cells are typically almost always included in whole-islet Ca^2+^ waves, which implies that they play an important role in the mediation of intercellular signals. Since hub cells tend to have a larger number of direct neighbors and shorter phase lags in the wave-based network, their Ca^2+^ signals are more likely to be highly correlated to a larger number of other cells. Moreover, the above regularity is in line with our previous finding that hub cells tend to dissipate more energy (79). Interestingly, we also noted a tendency of hub cells being activated first when stimulated with 12 mM glucose, while under 8 mM glucose no such trend was observed (Fig. 3 *C*), which corroborates our previous findings, but on a larger data set (11). Nevertheless, the discrepancy observed between both stimulation levels indicates that the guidance of Ca^2+^ elevations and the mediation of intercellular signals are very complex processes that are influenced by the interplay of a wide variety of factors, such as the metabolic activity, local connectivity, and variations in gap junctional conductances, as well as by the variability of excitability profiles (7,42). To make matters even more complicated, many of these factors are known to be glucose dependent and change with time. These reasons might account for the apparent inconsistencies reported in the recent literature (35,44,54,59,63,65). It has been shown that specific subpopulations of β cells, whose metabolic fingerprints overlap with the characteristics of hub cells, can recruit a disproportionally high number of their neighbors (35,65), and that there exists an overlap between the cells that drive glucose-stimulated Ca^2+^ elevations and hub cells (59). In contrast, it has also been reported that the first responding cells that guide the first responses to stimulation are defined principally by their excitability profiles and that they do not spatially or functionally overlap with hub cells (44), and similar conclusions have been drawn from comprehensive in silico studies (63).

While the connection between hub and first responder cells does not seem to be entirely clear, it is becoming apparent that the hub cells are not the cells that impose the cellular rhythm in the phase of sustained activity. Our results show that hubs are definitely not specialized initiators of intercellular waves (or pacemaker cells as also sometimes called in the literature). Specifically, there seems to be no correlation between the number of functional connections and the wave initiation parameter in 8 mM glucose, while in 12 mM glucose there was even a slight anticorrelation (Fig. 3 *D*), and there was only a random overlap between these two subpopulations (Fig. 5 *B*). These findings are also in agreement with a recent report on human islets (43) and with results predicted by a multicellular computational β cell model (63). However, even though the hub cells do not initiate Ca^2+^ waves, they could play an important role in the coordination of intercellular signals. In our analyses we compared their roles in the functional correlation-based network and in wave-derived networks and detected a strong relation between the number of connections in both types of networks, thereby highlighting their exceptional role in mediating the collective activity. Most importantly, a strong trend of hub cells exhibiting the shortest delays in transmitting the intercellular signal to their neighbors was detected in both glucose concentrations. This implies a higher level of intercellular coupling, which might be one of the crucial aspects of their decisive role in coordinating Ca^2+^ oscillations across the islets. What we call intercellular coupling essentially reflects the local efficiency of spreading depolarization and Ca^2+^ waves and has been termed this way in classical electrophysiological studies (82,83). This local spreading not only depends on the intercellular conductance determined by gap junctions but also on the capacitance and nonjunctional membrane conductance of the neighboring cells that are being depolarized by junctional currents (70). While the cell capacitance was estimated to contribute only marginally to the time lag between neighboring cells (70), the junctional and nonjunctional conductance are not only important but also change periodically between bursts and silent phases depending on a cell’s state of activation (82,83). We chose the term intercellular coupling since it is impossible in our current experiments to separately quantify the contributions by the junctional and nonjunctional conductance. Previous modeling suggested that K_ATP_ conductance of neighboring cells may determine the wave velocity more importantly than gap junctional conductance at the beginning of β cell bursting immediately following activation by glucose (84). Later during the sustained plateau phase the degree of synchronicity is higher and all cells are close to their thresholds for firing a burst when being activated by the incoming depolarization front, and thus the speed of the intercellular wave may be predominantly proportional to the square root of the harmonic mean of gap junctional conductivity (85). This theory also predicts that the wave velocities should be largely independent of the stimulatory glucose concentration, and we confirmed this (Fig. S1). Furthermore, the range of average node weights in this study was consistent with a wave velocity in the range of 30–100 *μ*m/s, which is comparable with previous experimental studies (39,64,75,84). Notably, the absolute values of wave velocities tend to be higher in modeling studies, unless a heterogeneity in intrinsic cell parameters or gap junctional coupling or both are introduced (85,86). In hub cells, the delays were approximately 20% shorter and, together with a larger number of neighbors, this could importantly contribute to the higher correlation of their Ca^2+^ signals with signals in other cells. In addition to the above circumstantial arguments about the importance of gap junctional coupling, to account for systematically higher local wave velocities between a hub cell and its neighbors across a large number of waves and wave directions going through it over tens of minutes, a larger gap junctional conductance of the observed hub cell could be a more plausible explanation than a significantly higher input resistance or state of activity in all of its neighbors from a probabilistic point of view, in the spirit of the Occam’s razor. However, this remains to be clarified in future studies.

To identify Ca^2+^ wave initiators and to assess intercellular interactions more precisely, we made use of the multilayer network formalism and regarded each calcium wave as an individual network layer with weighted directed connections portraying the intercellular signal propagation. We identified β cell subpopulations that act as wave initiators, and these cells represented the most active cells in the islet (Figs. 4 *A* and 5 *B*). This observation goes well in hand with previous studies in which the cells that lead Ca^2+^ oscillations were indirectly shown to have a faster intrinsic oscillation frequency, consistent with a rhythmic pacemaker concept (65,74). As such, the wave initiator population has been argued to be important for proper regulation of pulsatile insulin release during the second phase (59,65,74). In addition, this cell population was also found to have elevated excitability levels (13,74,75) and lower metabolic rates as they seem to exhibit lower NAD(P)H responses (65) and therefore differ in this respect from the hub cell population, in which an enhanced metabolic activity was noticed (35,46,81). Furthermore, we observed a rather small overlap between the subpopulation of cells that act as wave initiators and the cells responding first to initial exposure to stimulatory glucose concentration (Fig. 5 *B*). In addition, a clear tendency between the activation delay and the wave initiation parameter was only observed in 8 mM and not in 12 mM glucose (Fig. 4 *C*). On one hand this might be due to the abrupt cellular activations provoked by supraphysiological stimulation levels, but on the other hand this result once again indicates that the response to stimulation encompasses a series of complex factors, as already argued above. Furthermore, the cells that recurrently initiate Ca^2+^ waves have an average number of connections in the correlation-based networks and are thus not hubs. (Fig. 4 *D*). Our observation that wave initiators seem to have a lower-than-average number of connections may intuitively be ascribed to the fact that the waves start in these cells and thus they necessarily have fewer links in the wave-based network (with no cells preceding them). However, it is also consistent with previous suggestions that the cells with less neighbors may first be able to escape the hyperpolarizing or clamping effect of nonactive cells (74,87). Judging by the node weights, once the waves are established, compared with other cells in the wave-based network, the local intercellular coupling between wave initiators and their direct neighbors does not seem to be higher or lower than the average.

Two important questions in islet biology that remain open are whether heterogeneous nearest-neighbor coupling is sufficient to explain the patterns of observed intercellular waves and small-world functional networks properties and whether the behavior observed in a single focal plane is representative of the behavior in all three dimensions. Cappon and Pedersen demonstrated that structural long-range connections are not necessary for the observed intercellular waves and small-world functional network properties and that the latter can arise from heterogeneous nearest neighbor coupling of heterogeneous cells that are synchronized by heterogeneous propagating waves (61). Indeed, possible structural long-range connections typically prevent propagating waves (88) and the combination of experimentally observed intercellular waves and small-world functional network properties speaks against a structural small-world substrate. Notably, further research also suggested that the small-world characteristics can in part be attributed to the multimodal nature of the oscillatory β cell activity, whereby the slow oscillatory component contributes more directly to long-range connectivity (54) and that the slow-activity-derived networks are less dependent on the structural gap junctional network (62,91). Furthermore, both modeling and experimental studies suggest that the intercellular waves and functional networks in a single focal plane are largely representative of the behavior in the islet as a whole (59,61,64). Clearly, further studies are needed to address these two questions into more detail.

Furthermore, our results display a rather high dispersion of data. Individual data points are profoundly scattered across the main trends, which not only indicates that β cells are characterized by a large functional heterogeneity but also that the division to specific subpopulations cannot be done definitively as there is certainly some overlap. In other words, there are, for example, also some hub or wave-initiator cells with a lower-than-average activity, although the main trend clearly indicates that the cells with many functional connections or the cells that frequently initiate waves have on average higher active times. Most importantly, due to the heterogeneous nature of data, we gave particular emphasis to the statistical measures and interpretation of our results. Typically, the correlations between parameters have been studied and interpreted based solely on the statistical significance (p value) that shows only whether an effect exists and does not reveal the size of the effect (substantive significance). A major disadvantage of the p value is the dependency on the sample size. With a sufficiently large sample, as in our study, the p value will almost always show a significant difference, which can bring confusion to the interpretation of the results. As in our analyses we dealt with a high number of rather scattered data points; we additionally evaluated the results in terms of the effect size (δ value), which is independent of sample size and helps us to better understand the magnitude of the p value difference found (89). By these means we identified which correlations between parameters are physiologically meaningful despite the high degree of variability. To gain further insight into the data dispersion, we additionally investigated how the central Ca^2+^ signaling parameters depend on the node degrees and initiation parameters in individual islets. The results presented in Figs. S8 and S9 indicate that in most islets the same trends are noticed as in the main results with a few minor exceptions. Apparently, the dispersion of data points that characterize the results is more due to the intercellular variability than due to interislet variability.

Finally, representing each Ca^2+^ wave as a network layer with weighted directed connections not only enabled us to portray the intercellular signal propagation but also allowed us to assess the spatiotemporal stability of calcium waves (48). It turned out that subregions exist in the islet that serve as initiators in a large portion of events, but the course of the Ca^2+^ waves were found to change with time, whereby the changes were more frequent at a physiological glucose concentration (Fig. 6 *D*). We argue that this reflects the fact that, under high glucose conditions, i.e., 12 mM, the supply of metabolic energy is high, all cells are on average more excited, and the relative cell-to-cell variability becomes smaller, which facilitates a more stable course of intercellular signals. On the other hand, if the supply of metabolic energy is moderate, i.e., in 8 mM glucose, the cells remain less excited, more heterogeneous, and are therefore more prone to stochastic effects and influences from neighboring cells, resulting in less coherent spatiotemporal activity patterns. These ideas are also in agreement with recent theoretical studies that indicate that presence of wave initiators in networks of excitable cells as an emergent and dynamic population behavior (8,13,90). Notably, applying an equivalent analysis for the persistency of hub cells revealed that their role is much more stable in time, as particularly the number of functional connections of the most connected cells was not found to change significantly during the recordings (Fig. 6, *C* and *E*). Moreover, in contrast to wave-initiator cells, the persistency of hub cells did not depend on glucose concentration. These results suggest that the role of hub cells might be more predetermined by their intrinsic functional characteristics (such as their metabolic activity and the degree of intercellular coupling) and less on the level of stimulation compared with wave-initiator cells. In future studies, it would be worthwhile to assess the stability of the hub role over longer periods of time, e.g., several hours or even days, and to reassess if this stability is related to the cell maturation stage and other functional properties by analyzing expression of different transcription factors.

In sum, in the present work we assess multicellular activity in pancreatic islets with emphasis on specialized subpopulations of β cells, which substantially affect the collective dynamics. Our findings indicate that both hub and wave-initiator cells are genuine features of islets, but they differ in several aspects of Ca^2+^ signaling and their roles do not seem to overlap. Moreover, while on the molecular and single-cell level clear discrepancies have been identified, suggesting differences in function (7,31,52), it appears that the roles of cells, when operating in a multicellular environment, are not completely obvious and predetermined. Particularly the initiation of intercellular waves was found to be rather dynamic and is most probably affected by a wide range of factors. It seems that a higher fraction of cells exhibits the potential to become a wave initiator, but the underlying socio-cellular context then principally defines the true roles. This idea is also in agreement with recent theoretical studies highlighting that pacemakers (in the context of wave initiators) can emerge naturally in cellular networks (90). Finally, we did not perform silencing or deletion of wave-initiating cells but, given the rather large number of physically separate cell clusters capable of initiating waves found in our study, it seems reasonable to speculate that silencing or deleting some of them would probably not abolish intercellular waves. The role of hub cells seems to be more determined, whereby a higher degree of intercellular coupling along with the specific metabolic characteristics are the main determinants that ensure their more stable roles. Importantly, our results do not indicate that the roles of hub cells are reserved exclusively to a very small fraction, i.e., a few extraordinary and irreplaceable cells. Rather, we argue that, in the heterogeneous β cell population, a certain fraction of cells possesses more exceptional values of certain electrophysiological and metabolic characteristics that are otherwise distributed continuously among cells. Therefore, they participate in the majority of Ca^2+^ waves, have the shortest delays to Ca^2+^ signals in other cells, and their oscillations are less variable. As a consequence, their signals are correlated with a large number of other cells and they emerge as hubs when viewed through the prism of network analysis. A similar concept has recently been proposed theoretically (51), unifying thereby the seemingly opposing views on β cell hubs (53,58). Furthermore, based on the rather weak and inconsistent overlap between different cell subpopulations reported here, as well as in the recent literature, we can presume that the functional heterogeneity in the β cell population exists for sure, but it is probably not as clear-cut as to divide cells into clearly predefined subgroups and that to some extent the influential cells can manifest themselves endogenously within the β cell collectives. We share the belief that such a design represents a functionally more robust and evolutionary advantageous architecture.

## Author contributions

M.Š., A.S., J.D., and M.G. conceived and designed the research. M.S.K., L.K.B., J.D., E.P.L., and J.K. performed the experiments. M.Š. and M.G. developed software and analyzed data. A.S., J.D., M.P., M.S.R., and M.G. interpreted results of the experiments. M.Š. prepared the figures. J.K. performed statistical analyses. M.G. and A.S. supervised the study. M.Š., J.D., and M.G. drafted the manuscript. M.Š., J.D., M.S.K., L.K.B., E.P.L., J.K., M.P., M.S.R., A.S., and M.G. edited and revised the manuscript and approved its final version.

## Supporting material

Document S1. Figures S1–S9

Document S2. Article plus supporting material
