# Supplementary material for: Functional characteristics of hub and wave-initiator cells in β cell networks
Source: Biophys J. 2023 Feb 2;122(5):784–801. doi: 10.1016/j.bpj.2023.01.039 (PMC10027448; doi:10.1016/j.bpj.2023.01.039)
Supplement: Document S1. Figures S1–S9 [file mmc1.pdf]

Biophysical Journal, Volume *122*

Supplemental information

Functional characteristics of hub and wave-initiator cells in β cell networks

Marko Šterk, Jurij Dolenšek, Maša Skelin Klemen, Lidija Križančić Bombek, Eva Paradiž Leitgeb, Jasmina Kerčmar, Matjaž Perc, Marjan Slak Rupnik, Andraž Stožer, and Marko Gosak

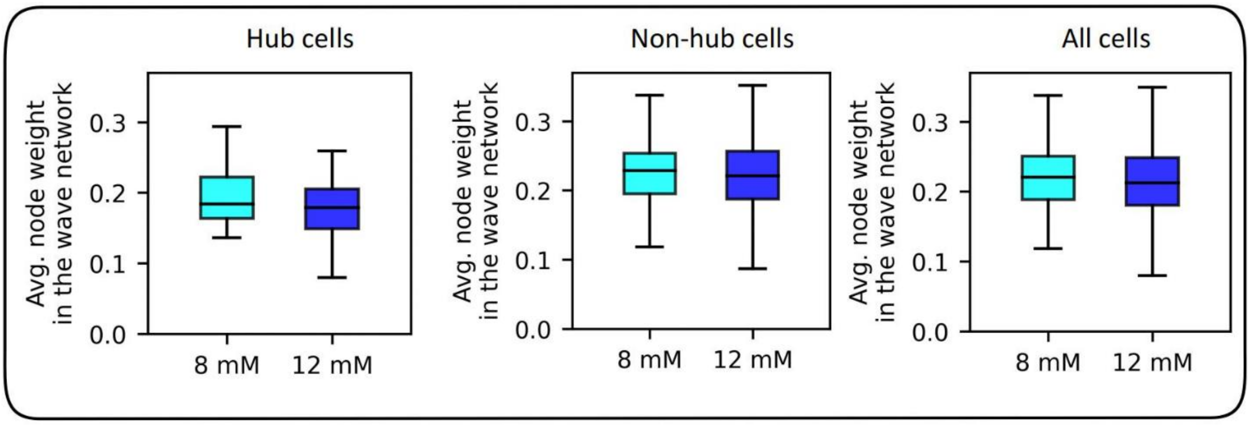

Figure S1. Absolute values of node weights in the wave network at 8 mM and 12 mM glucose concentration. Absolute values for hub cells (left panel), non-hub cells (middle panel) and all cells (right panel) in 8 mM (cyan) and 12 mM (blue) glucose. Hub cells were identified as 1/6 of cells with the highest number of functional connections. Boxes determine the interval between the 25th and the 75th percentile, whiskers denote the 10th and the 90th percentile, and lines within the boxes indicate the median. Data were pooled from the following number of islets/cells: 8/865 (8 mM glc); 8/1103 (12 mM glc).

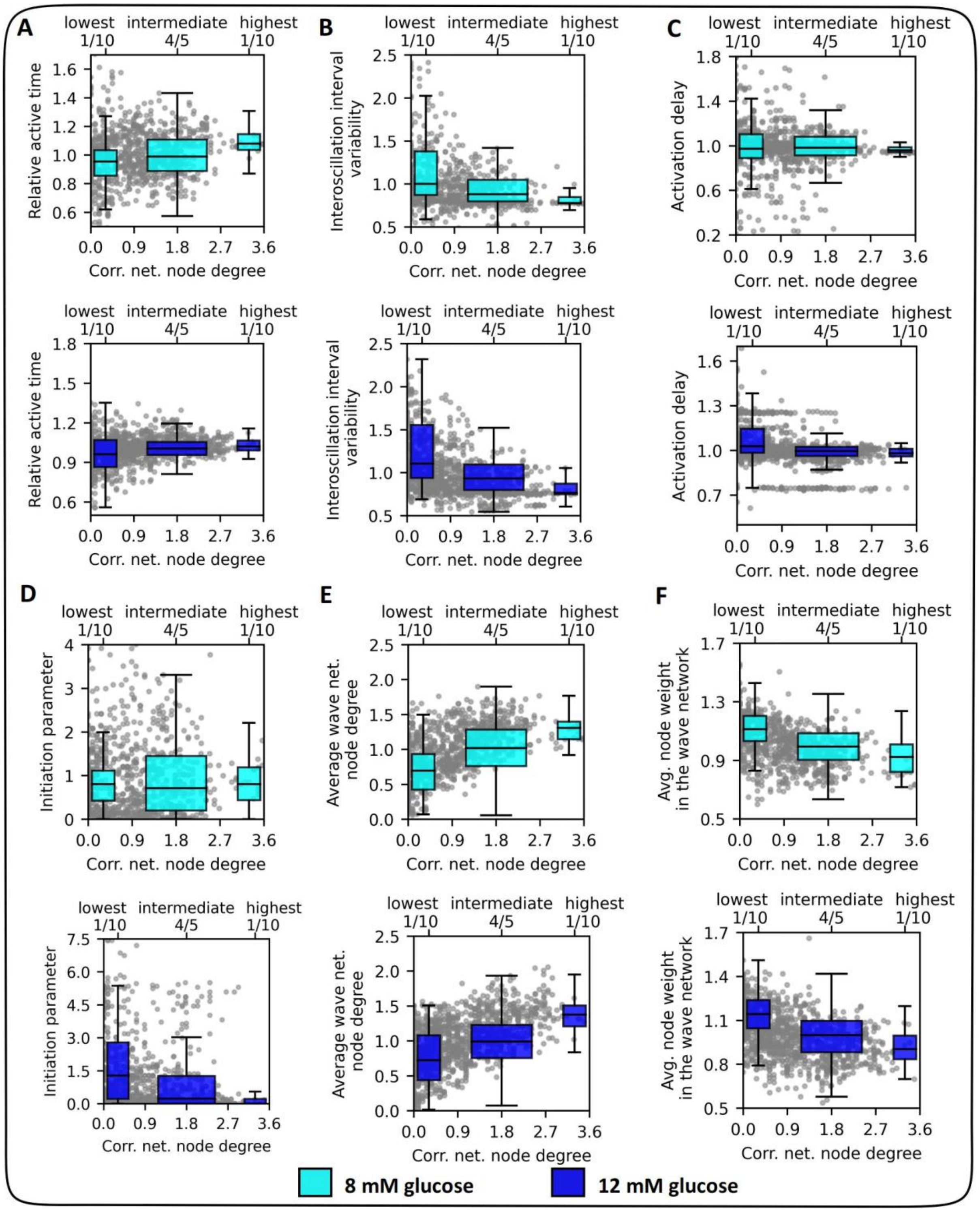

Figure S2. Relationship between various Ca^{2+} signaling parameters and the number of functional connections in correlation-based networks. Dependence of the relative active time **(A)**, inter-oscillation interval variability **(B)**, activation delay **(C)**, initiation parameter **(D)**, wave-network node degree **(E)**, and the node weight in the wave network, i.e., average transmission delay **(F)** on the correlation network degree for stimulation protocols with 8 mM glucose (first and third row, cyan) and 12 mM glucose (second and fourth row, blue). Grey dots represent normalized values of individual cells, and box plots show the lowest 1/10, the middle 4/5, and the highest 1/10 connected cells in the functional network. All panels show the pooled data from all recordings whereby individual values were normalized by the average value of the specific parameter in the given islet. Boxes determine the interval between the 25th and the 75th percentile, whiskers denote the 10th and the 90th percentile, and lines within the boxes indicate the median. Data were pooled from the following number of islets/cells: 8/865 (8 mM glc); 8/1103 (12 mM glc).

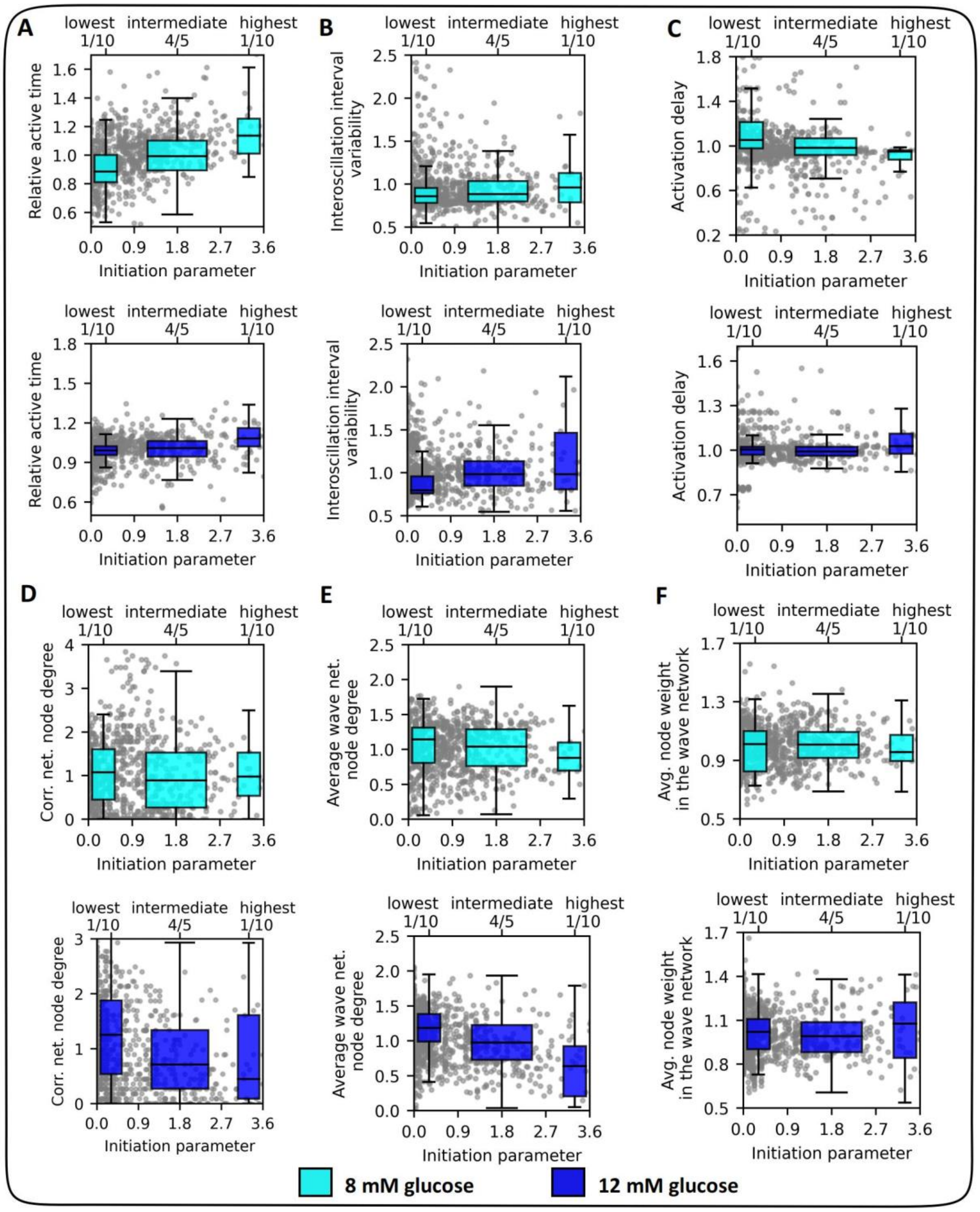

Figure S3. Relationship between various Ca^{2+} signaling parameters and the wave initiation parameter. Dependence of the relative active time **(A)**, inter-oscillation interval variability **(B)**, activation delay **(C)**, correlation-based network node degree **(D)**, wave-network node degree **(E)**, and the node weight in the wave network **(F)** on the initiation parameter for stimulation protocols with 8 mM glucose (first and third row, cyan) and 12 mM glucose (second and fourth row, blue). Grey dots represent values of individual cells, and box plots show the lowest 1/10, the middle 4/5, and the highest 1/10 cells based on their initiation parameter. All panels show the pooled data from all recordings whereby individual values were normalized by the average value of the specific parameter in the given islet. Boxes determine the interval between the 25th and the 75th percentile, whiskers denote the 10th and the 90th percentile, and lines within the boxes indicate the median. Data were pooled from the following number of islets/cells: 8/865 (8 mM glc); 8/1103 (12 mM glc).

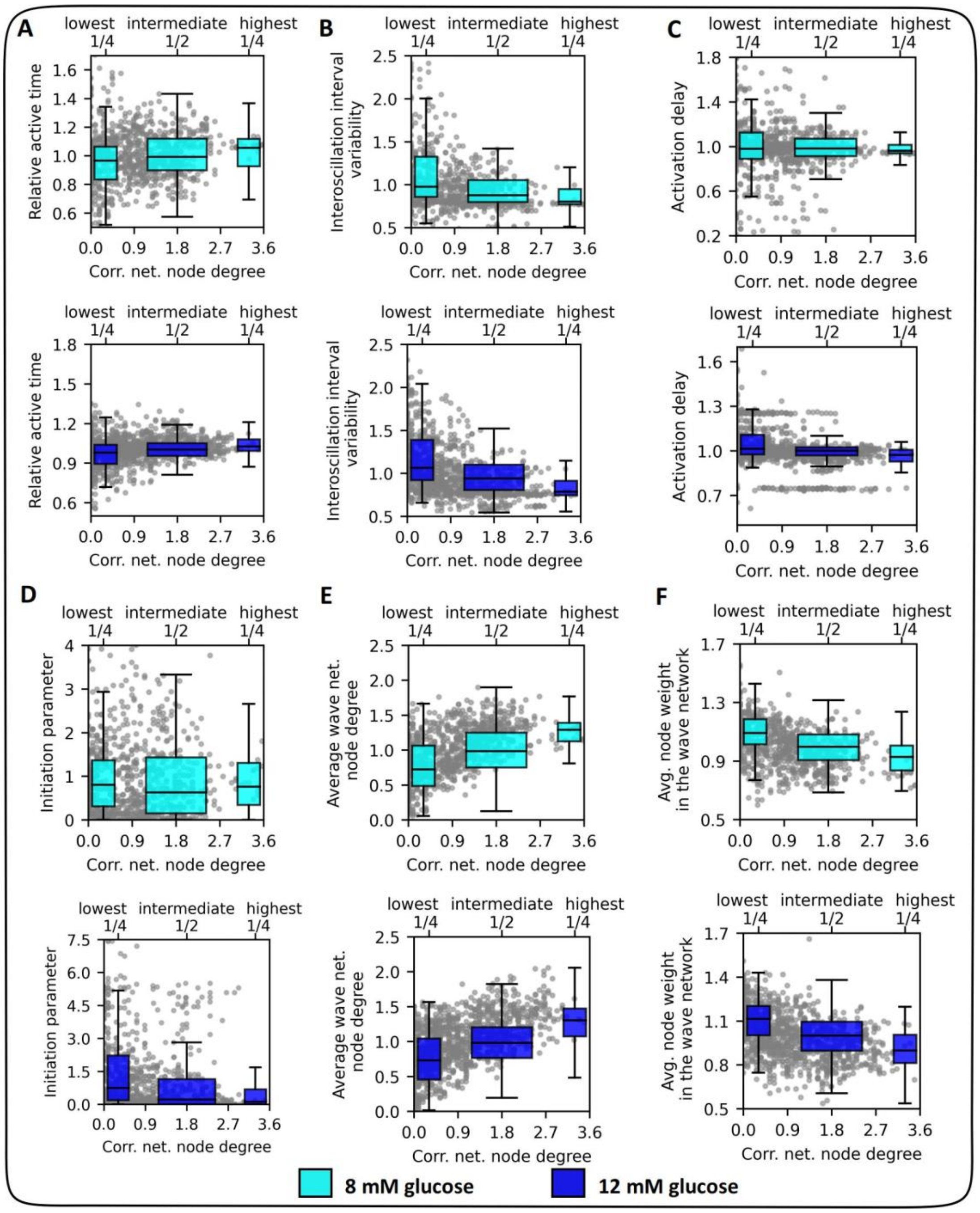

Figure S4. Relationship between various Ca^{2+} signaling parameters and the number of functional connections in correlation-based networks. Dependence of the relative active time **(A)**, inter-oscillation interval variability **(B)**, activation delay **(C)**, initiation parameter **(D)**, wave-network node degree **(E)**, and the node weight in the wave network, i.e., average transmission delay **(F)** on the correlation network degree for stimulation protocols with 8 mM glucose (first and third row, cyan) and 12 mM glucose (second and fourth row, blue). Grey dots represent normalized values of individual cells, and box plots show the lowest 1/4, the middle 1/2, and the highest 1/4 connected cells in the functional network. All panels show the pooled data from all recordings whereby individual values were normalized by the average value of the specific parameter in the given islet. Boxes determine the interval between the 25th and the 75th percentile, whiskers denote the 10th and the 90th percentile, and lines within the boxes indicate the median. Data were pooled from the following number of islets/cells: 8/865 (8 mM glc); 8/1103 (12 mM glc).

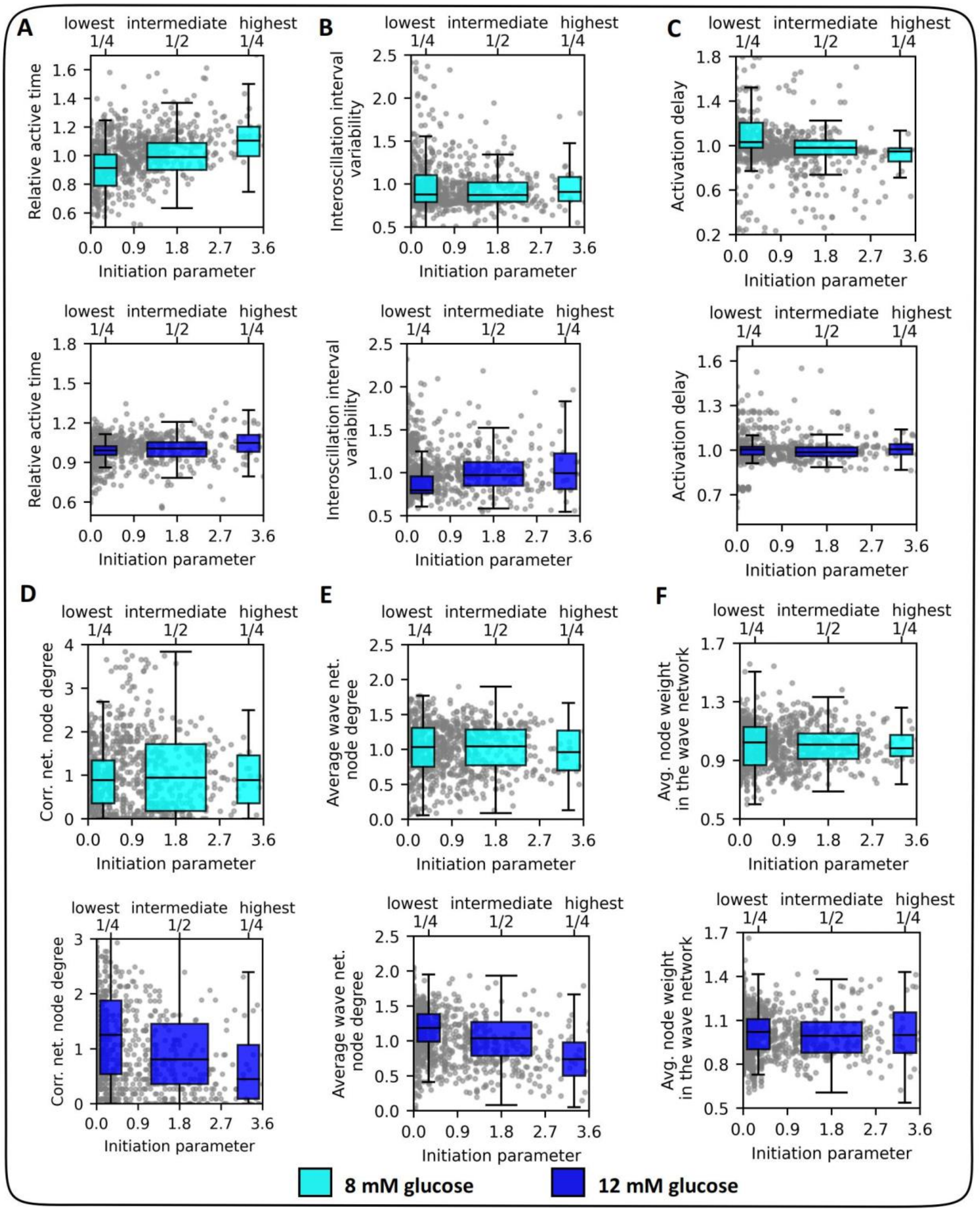

Figure S5. Relationship between various Ca^{2+} signaling parameters and the wave initiation parameter. Dependence of the relative active time **(A)**, inter-oscillation interval variability **(B)**, activation delay **(C)**, correlation-based network node degree **(D)**, wave-network node degree **(E)**, and the node weight in the wave network **(F)** on the initiation parameter for stimulation protocols with 8 mM glucose (first and third row, cyan) and 12 mM glucose (second and fourth row, blue). Grey dots represent values of individual cells, and box plots show the lowest 1/4, the middle 1/2, and the highest 1/4 cells based on their initiation parameter. All panels show the pooled data from all recordings whereby individual values were normalized by the average value of the specific parameter in the given islet. Boxes determine the interval between the 25th and the 75th percentile, whiskers denote the 10th and the 90th percentile, and lines within the boxes indicate the median. Data were pooled from the following number of islets/cells: 8/865 (8 mM glc); 8/1103 (12 mM glc).

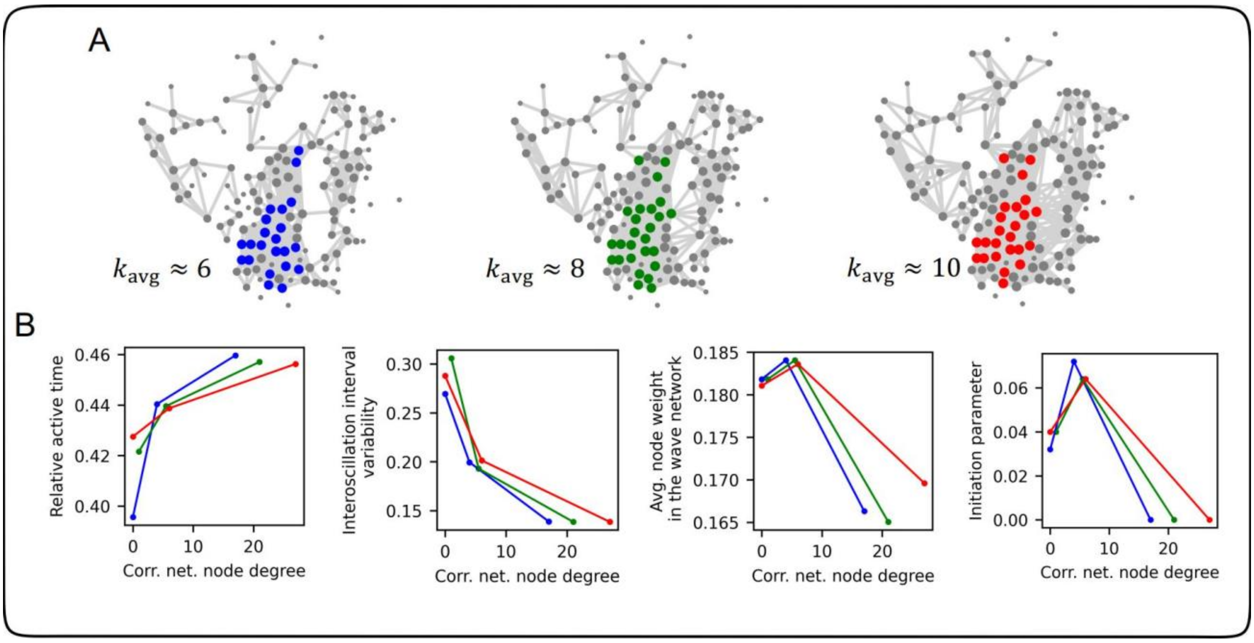

Figure S6. Sensitivity of various Ca^{2+} signaling parameters on the selected average correlation network node degree. A) Three correlation-based functional networks constructed from Ca^{2+} activity within the same islet with average node degrees $k_{avg} = 6$ (left), $k_{avg} = 8$ (middle) and $k_{avg} = 10$ (right). Dots represent physical locations of cells and the colors blue, green and red represent identified hub cells (top 1/6 of most connected cells) in each network. **B)** Relative active time, inter-oscillation interval variability, average node weight in the wave network, and initiation parameter as a function of the correlation network node degree for the three different values of k_{avg}. Dots signify the 1/6 lowest, 2/3 intermediate and 1/6 cells with the highest number of functional connections for different average network degrees (colors of dotted lines correspond to the color-coding in in panel A).

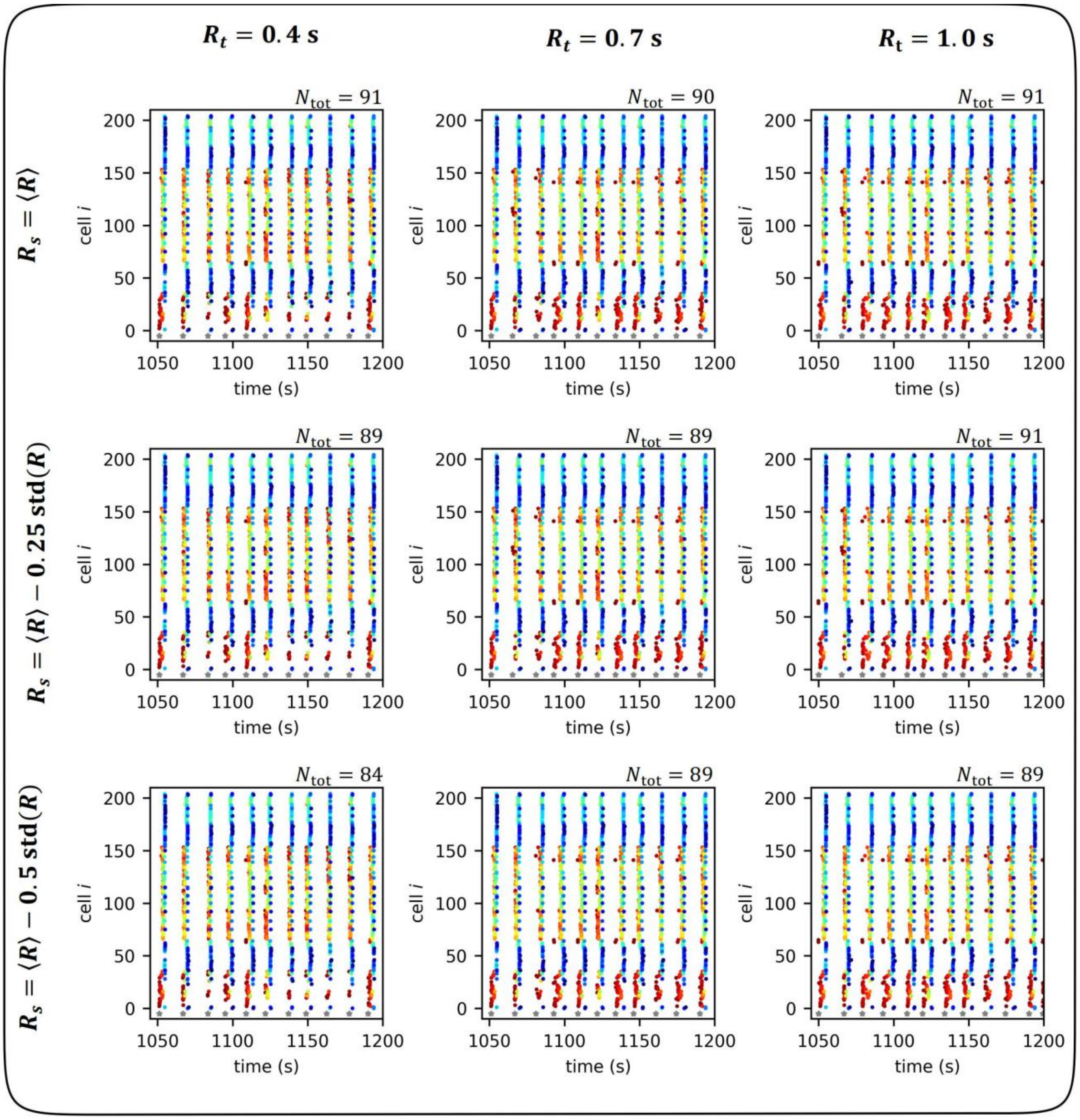

Figure S7. Sensitivity analysis of detected waves in a representative islet with regard to the wave detection threshold parameters. Outtakes of raster plots of cellular activation sequences of detected waves for different distance (R_s) and temporal (R_t) threshold parameters. Colored dots represent the oscillation onset time (time of activation) of individual cells within individual detected waves. Red color indicates cells that activated first, and blue color indicated cells that activated last in a given wave. N_{tot} denotes the total number of detected waves in the whole plateau phase in the islet. R_s is calculated based on the average intercellular distance <*R*> within the given islet and the corresponding standard deviation std(*R*). Evidently, the waves and the corresponding cellular activation sequences are very similar in all panels, indicating thereby that the wave detection algorithm is not sensitive to the choice of parameters.

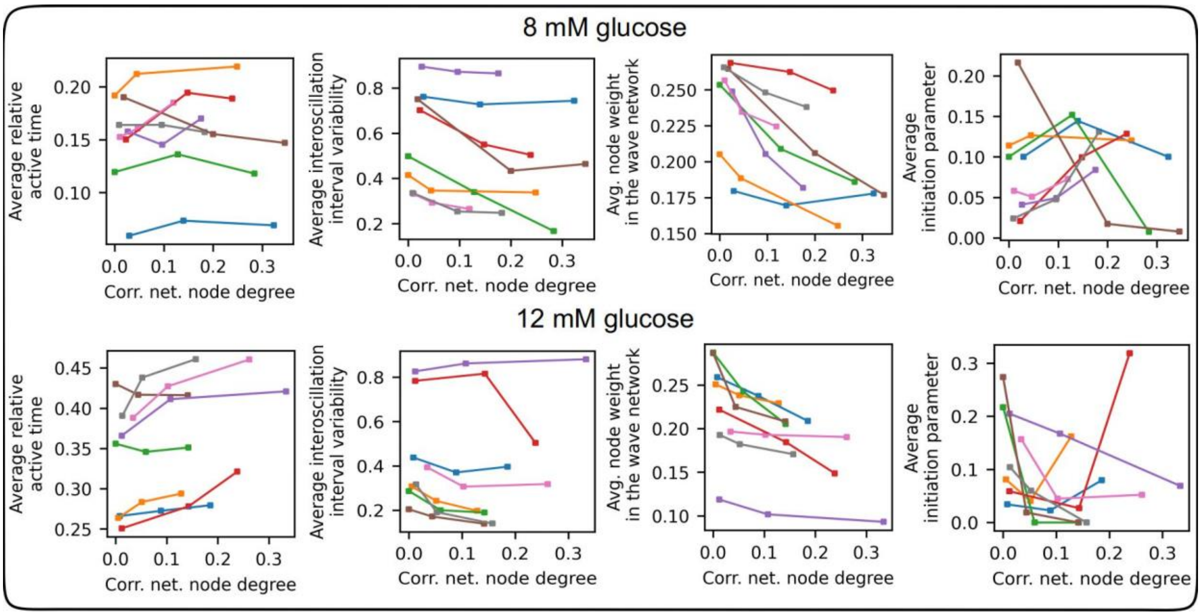

Figure S8. Relationship between absolute values of various Ca^{2+} signaling parameters and the correlation network node degrees in individual islets. Average relative active time (first column), average inter-oscillation interval variability (second column), average node weight in the wave network (third column), and average initiation parameter (fourth column) as a function of the correlation network node degree for different islets (indicated with different colors) and for both stimulation protocols (8 mM glucose – top row, 12 mM glucose – bottom row). Dots represent cell groups with the 1/6 lowest, 2/3 intermediate and 1/6 highest correlation network node degrees in each recording.

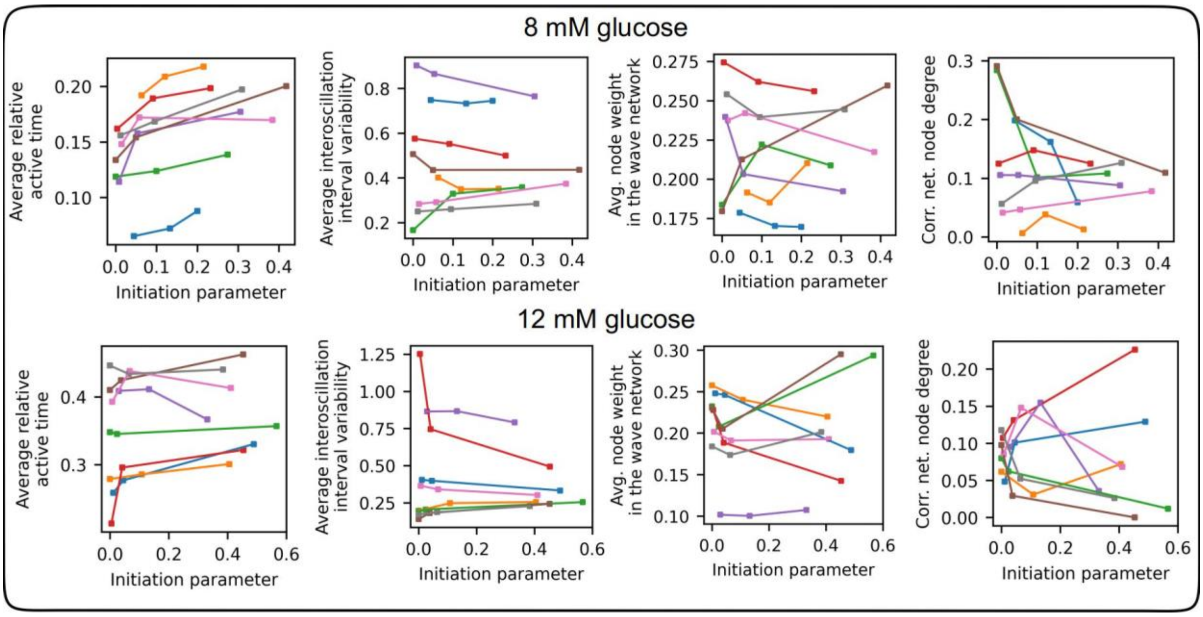

Figure S9. Relationship between absolute values of various Ca^{2+} signaling parameters and the initiation parameter in individual islets. Average relative active time (first column), average inter-oscillation interval variability (second column), average node weight in the wave network (third column), and correlation network node degree (fourth column) as a function of the initiation parameter for different islets (indicated with different colors) and for both stimulation protocols (8 mM glucose – top row, 12 mM glucose – bottom row). Dots represent cell groups with the 1/6 lowest, 2/3 intermediate and 1/6 highest initiation parameter in each recording.
